# Monitoring metal–amyloid-β complexation by a FRET-based probe: design, detection, and inhibitor screening†
†Electronic supplementary information (ESI) available: Experimental section, Table S1, and Fig. S1–S10. See DOI: 10.1039/c8sc04943b


**DOI:** 10.1039/c8sc04943b

**Published:** 2018-12-06

**Authors:** Hyuck Jin Lee, Young Geun Lee, Juhye Kang, Seung Hyun Yang, Ju Hwan Kim, Amar B. T. Ghisaidoobe, Hyo Jin Kang, Sang-Rae Lee, Mi Hee Lim, Sang J. Chung

**Affiliations:** a Department of Chemistry , Korea Advanced Institute of Science and Technology (KAIST) , Daejeon 34141 , Republic of Korea . Email: miheelim@kaist.ac.kr; b Department of Chemistry , Dongguk University , Seoul 04620 , Republic of Korea . Email: jin0305@dongguk.edu; c Department of Chemistry , Ulsan National Institute of Science and Technology (UNIST) , Ulsan 44919 , Republic of Korea; d School of Pharmacy , Sungkyunkwan University , Suwon 16419 , Republic of Korea . Email: sjchung@skku.edu; e National Primate Research Center (NPRC) , Korea Research Institute of Biosience and Biotechnology , Cheongju , Chungbuk 28116 , Republic of Korea . Email: srlee@kribb.re.kr

## Abstract

Aggregation of amyloidogenic peptides could cause the onset and progression of neurodegenerative diseases, such as Alzheimer's disease and Parkinson's disease. These amyloidogenic peptides can coordinate to metal ions, including Zn(ii), which can subsequently affect the peptides' aggregation and toxicity, leading to neurodegeneration. Unfortunately, the detection of metal–amyloidogenic peptide complexation has been very challenging. Herein, we report the development and utilization of a probe (**A-1**) capable of monitoring metal–amyloid-β (Aβ) complexation based on Förster resonance energy transfer (FRET). Our probe, **A-1**, is composed of Aβ_1–21_ grafted with a pair of FRET donor and acceptor capable of providing a FRET signal upon Zn(ii) binding even at nanomolar concentrations. The FRET intensity of **A-1** increases upon Zn(ii) binding and decreases when Zn(ii)-bound **A-1** aggregates. Moreover, as the FRET intensity of Zn(ii)-added **A-1** is drastically changed when their interaction is disrupted, **A-1** can be used for screening a chemical library to determine effective inhibitors against metal–Aβ interaction. Eight natural products (out of 145 compounds; >80% inhibition) were identified as such inhibitors *in vitro*, and six of them could reduce Zn(ii)–Aβ-induced toxicity in living cells, suggesting structural moieties useful for inhibitor design. Overall, we demonstrate the design of a FRET-based probe for investigating metal–amyloidogenic peptide complexation as well as the feasibility of screening inhibitors against metal-bound amyloidogenic peptides, providing effective and efficient methods for understanding their pathology and finding therapeutic candidates against neurodegenerative disorders.

## Abstract 2

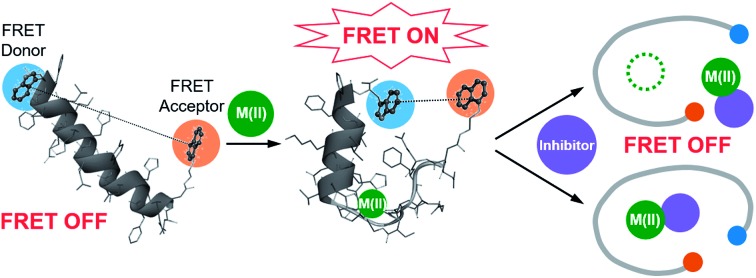
A FRET-based method was developed for monitoring metal–amyloid-β complexation and identifying inhibitors against such interaction.

## Introduction

**Fig. 1:**
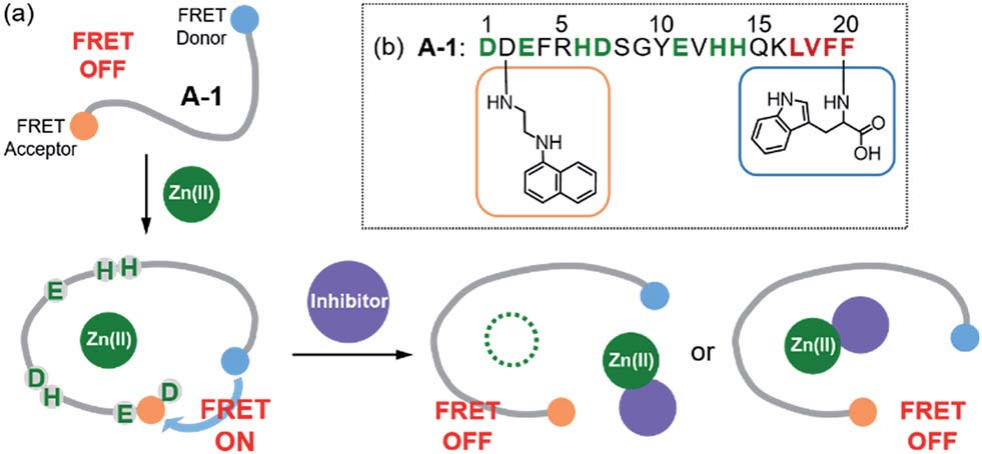
Design principle and sequence of the FRET-based probe, **A-1**. (a) FRET responses of **A-1** in the absence and presence of Zn(ii) with and without inhibitors. (b) Amino acid sequence of **A-1**. **A-1** is composed of Trp (blue box) at the C-terminus as a FRET donor and 1-naphthylethylenediamine conjugated to the side chain of the Asp (orange box) at the N-terminus as a FRET acceptor. Proposed amino acid residues for metal binding and a portion of the self-recognition site are indicated in green and red, respectively.

**Scheme 1:**
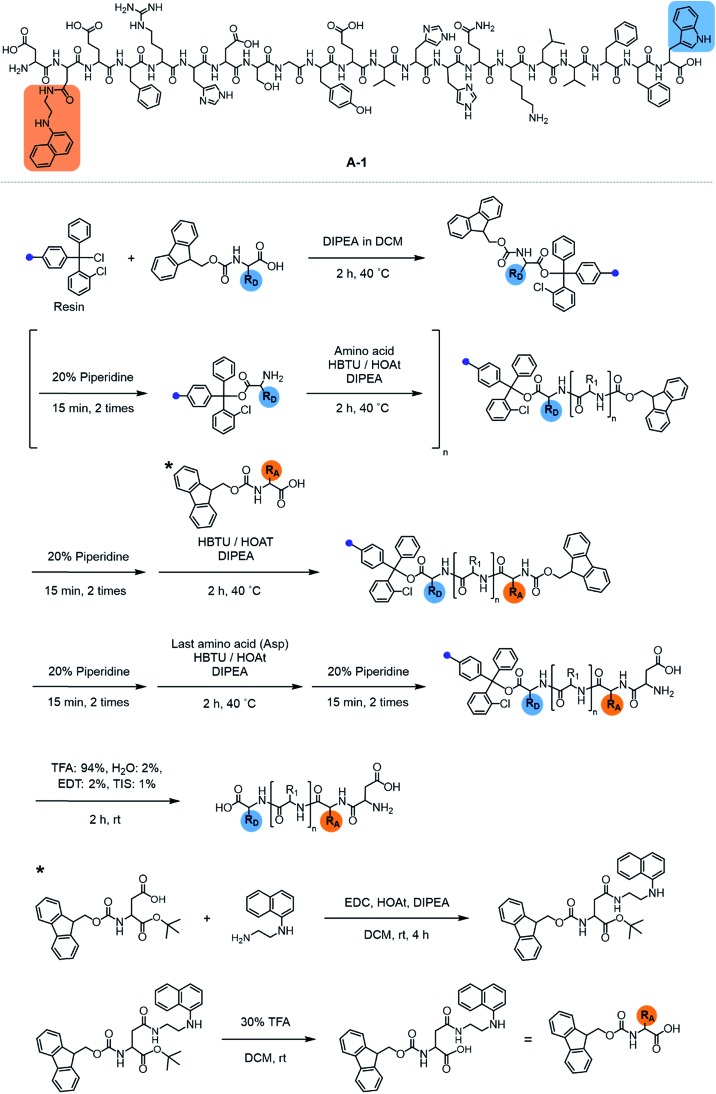
Synthetic routes to **A-1**.

The number of aged people affected by neurodegenerative diseases has been increasing; however, the development of treatments for the diseases has not been successful due to the lack of understanding about their pathogenesis.^1^,^2^ The proposed risk factors of neurodegenerative diseases include metal ions [*e.g.*, Zn(ii)] and amyloidogenic peptides [*e.g.*, amyloid-β (Aβ) and tau for Alzheimer's disease, α-synuclein for Parkinson's disease, and huntingtin for Huntington's disease].^3^–^12^ Toxic aggregates are formed upon aggregation of these amyloidogenic peptides, particularly in the presence of metal ions.^2^,^13^–^15^ The aggregation and conformational changes of such amyloidogenic peptides have been previously studied by luminescence, including Förster resonance energy transfer (FRET).^16^–^21^ In addition, the interactions between amyloidogenic peptides and metal ions (*e.g.*, binding affinity and coordination geometry) have been investigated through multiple physical methods.^8^,^9^,^22^–^26^ Such approaches, however, require high concentrations of peptides and metal ions (*e.g.*, high μM) presenting significant challenge in performing the experiments due to the aggregation-prone properties of amyloidogenic peptides. Unfortunately, detecting the formation of metal-bound amyloidogenic peptides with a straightforward and efficient method (*e.g.*, monitoring a turn-on signal) at a low concentration (*ca.* nM) has not been reported. Herein, we report a FRET-based probe (**A-1**; Fig. 1 and Scheme 1), composed of Aβ_1–21_ grafted with a pair of FRET donor and acceptor, for monitoring metal–Aβ complexation at a nanomolar range with a turn-on FRET signal. The FRET intensity of **A-1** was observed to increase upon binding to Zn(ii) (green; Fig. S1†). Note that although other metal ions [particularly, Cu(ii)] are reported to interact with Aβ,^10^,^24^ the use of our probe, **A-1**, is limited for paramagnetic metal ions, such as Cu(ii), because its fluorescence is quenched (Fig. S1†). Additionally, the FRET signal of **A-1** was changed when (i) Zn(ii) binding of **A-1** was interfered by the metal chelator, **EDTA** (ethylenediamine tetraacetic acid),^29^ or the compound, **L2-b** [*N*^1^*N*^1^-dimethyl-*N*^4^-(pyridin-2-ylmethyl)benzene-1,4-diamine],^30^,^31^ capable of forming a ternary complex with Zn(ii)–Aβ; (ii) the probe was aggregated. Moreover, a library of natural products as inhibitors against metal–Aβ interaction was screened based on the change in the FRET responses of Zn(ii)-treated **A-1**. 8 out of 145 natural products were identified as effective inhibitors (>80% inhibition) *in vitro*. Among the 8 molecules, 6 compounds were shown to lower the toxicity associated with Zn(ii)–Aβ in living cells. Our studies demonstrate the feasibility of developing an efficient tactic to probe metal–amyloidogenic peptide complexation, along with its potential as a screening tool for drug discovery against neurodegenerative diseases.

## Results and discussion

### Design and preparation of **A-1**

Our probe, **A-1**, was designed to have a FRET donor (Trp; *λ*_ex_ = 280 nm, *λ*_em_ = 350 nm) and an acceptor (1-naphthylethylenediamine conjugated to the side chain of an Asp; *λ*_ex_ = 350 nm, *λ*_em_ = 420 nm) for FRET at the C- and N-termini of the Aβ_1–21_ sequence, respectively (Fig. 1). Aβ_1–21_ was selected as the main framework of **A-1** to include the metal binding site of Aβ (Fig. 1b; proposed metal binding residues highlighted in green, *e.g.*, Asp1, Glu3, His6, Asp7, Glu11, His13, and His14).^10^,^26^,^32^–^35^ Thus, **A-1** itself can interact with metal ions like Aβ. When **A-1** was treated with Zn(ii), the Zn(ii)–**A-1** complex was formed which was confirmed by mass spectrometry (MS) (Fig. S2†). Additionally, the binding affinity [*K*_d_ = 5.6 (±0.9) μM] of **A-1** (5 μM) for Zn(ii) was measured by a fluorescence measurement (Fig. S3a†), similar to the *K*_d_ values of Zn(ii)–Aβ obtained using the same method from previous studies.^36^–^38^ Moreover, the progression of peptide aggregation could be observed because **A-1** contains a portion of Aβ′s self-recognition site (Fig. 1b; red, Leu17–Phe20).^10^,^33^,^39^ **A-1** was synthesized through solid phase peptide synthesis. The detailed synthetic routes are described in Scheme 1 and Experimental section.†

### FRET signal of **A-1** upon binding to Zn(ii)

**Fig. 2:**
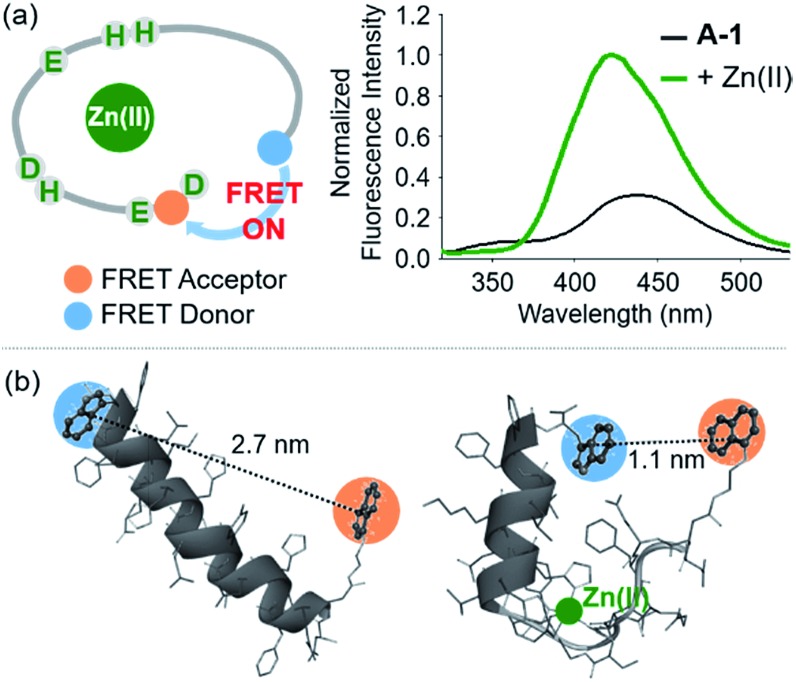
FRET response of **A-1** to Zn(ii) and proposed structures of metal-free and Zn(ii)-bound **A-1**. (a) Change in fluorescence upon incubation of **A-1** (black) with Zn(ii) (green). Conditions: [**A-1**] = 0.5 μM; [ZnCl_2_] = 100 μM; *λ*_ex_ = 280 nm. (b) Proposed structures of metal-free **A-1** (left) and Zn(ii)-bound **A-1** (right). The structures were generated by modifications of the previously reported structures of metal-free Aβ (PDB: ; 1AMC)^27^ and Zn(ii)-bound Aβ (PDB:; 1ZE9).^28^ The approximate distances between the FRET donor and acceptor were indicated with dashed lines.

**Fig. 3:**
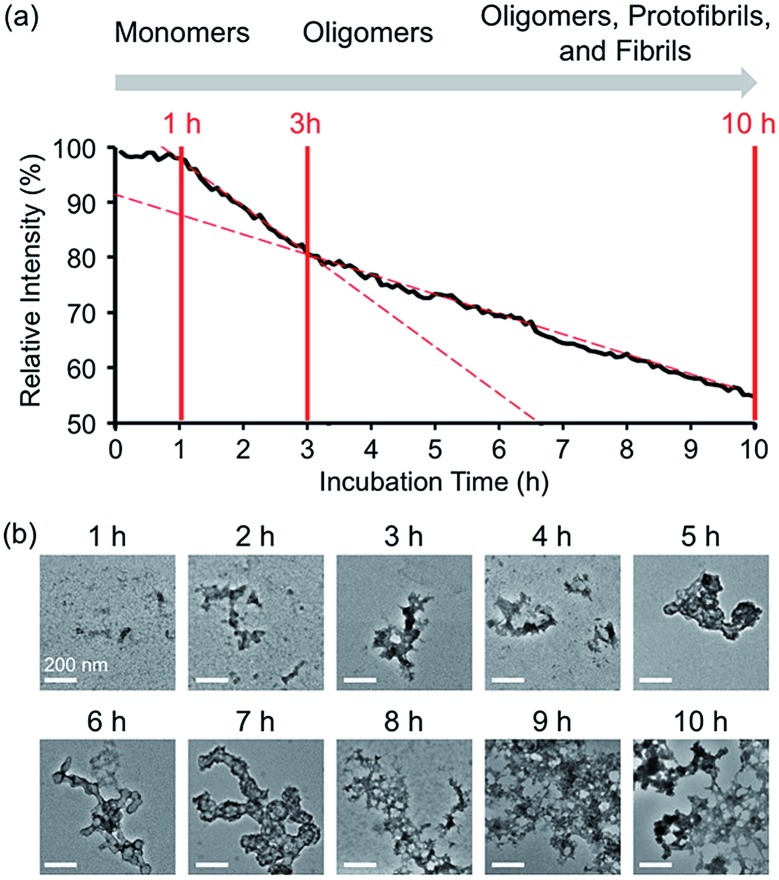
Time-dependent fluorescent response and aggregation progression of Zn(ii)-treated **A-1**. (a) Change in the FRET signal of **A-1** with Zn(ii) as a function of incubation time. (b) TEM images of Zn(ii)-added **A-1** aggregates generated at various incubation time points (scale bar = 200 nm). Conditions: [**A-1**] = 0.25 μM (for FRET) and 2.5 μM (for TEM); [ZnCl_2_] = 100 μM (for FRET) and 1 mM (for TEM); *λ*_ex_ = 280 mm; *λ*_em_ = 420 nm; incubation up to 10 h; room temperature.

The presence of Zn(ii) induced a significant turn-on FRET signal of **A-1** by >2 fold compared to Zn(ii)-free environment (Fig. 2a). In order to minimize the aggregation of Zn(ii)–**A-1** (*vide infra*; Fig. 3), along with consideration of our probe's Zn(ii) binding property, 250–500 nM of the probe and 100 μM of Zn(ii) were used for this study. As shown in Fig. S3b,† the fluorescence intensity of **A-1** (500 nM) at 420 nm was enhanced upon titration and was saturated at *ca.* 100 μM of Zn(ii). Since FRET occurs when a suitable donor and acceptor pair is in close proximity (1–10 nm) with the parallel orientation of the transition dipoles of the FRET donor and acceptor,^40^,^41^ an increase in the FRET intensity is indicative of **A-1**'s folding upon Zn(ii) binding (Fig. 2a). The possible conformations of metal-free and Zn(ii)-bound **A-1** were visualized by modeling with modifications of the previously reported structures of metal-free Aβ and Zn(ii)-bound Aβ (PDB: ; 1AMC 27 and ; 1ZE9,^28^ respectively; Fig. 2b). Without Zn(ii), although the indole ring of the FRET donor and the naphthalene ring of the FRET acceptor are close enough for energy transfer (*ca.* 2.7 nm), they are not facing each other and shown to be unfavorable to have a dipole–dipole interaction for FRET (Fig. 2b; left). Upon interacting with Zn(ii), however, the indole and naphthalene rings become closer (*ca.* 1.1 nm) than those in metal-free **A-1** and are facing each other which could be favorable for the dipole–dipole interaction necessary for energy transfer, suggesting that an efficient FRET signal could be observed upon Zn(ii) binding to the probe (Fig. 2b; right). Additionally, the emission spectrum was blue shifted by *ca.* 25 nm possibly due to an environmental change of the FRET acceptor, naphthylamine, when **A-1** was folded with Zn(ii) treatment (Fig. 2a; right). Note that we cannot rule out that intermolecular interactions resulted from **A-1**'s propensity to aggregate may induce the FRET.

### Aggregation of Zn(ii)-bound **A-1**

In the absence of Zn(ii), the FRET signal of **A-1** reduced as a function of incubation time (*ca.* 70% and *ca.* 85% decrease after 1 and 3 h incubation, respectively; Fig. S4†). This lowered signal may be triggered by the aggregation of **A-1** since the probe contains a portion of the self-recognition region of Aβ.^10^,^33^,^39^ In contrast, following incubation time, the FRET signal of Zn(ii)-treated **A-1** decreased (*ca.* 2% and *ca.* 18% decrease after 1 and 3 h incubation, respectively; Fig. 3a) at a slower rate compared to that of Zn(ii)-free **A-1** (Fig. S4†). This indicates that the aggregation of **A-1** could be delayed by the presence of Zn(ii), as observed with full-length Aβ_40_ (Fig. S5†). This difference could stem from the disparate conformations of Aβ aggregates generated upon the aggregation of metal–Aβ, distinct from those of metal-free Aβ aggregates.^28^,^42^ Thus, we analyzed the morphologies of Zn(ii)–**A-1** aggregates upon incubation by transmission electron microscopy (TEM). As depicted in Fig. 3b, small and amorphous aggregates were observed after 1 h incubation of Zn(ii)-added **A-1** followed by the detection of larger and more structured aggregates with longer incubation. Based on the variation of the FRET intensity as the probe aggregated, the aggregation process of Zn(ii)–**A-1** could be divided into three stages: (i) 0–1 h; (ii) 1–3 h; (iii) 3–10 h (Fig. 3a). Up to 1 h incubation, the FRET signal of Zn(ii)–**A-1** did not significantly decrease from the initial measurement. From 1 to 3 h, the FRET intensity of Zn(ii)–**A-1** dropped drastically and after 3 h incubation, the FRET responses of Zn(ii)–**A-1** were shown to be distinguishably reduced slower than those during the 1–3 h incubation period. This could be because Zn(ii)–**A-1** formed large-sized aggregates, including protofibrils and fibrils, which might restrict its rotation to limit the distance between the FRET donor and acceptor, along with its solubility in aqueous media. Thus, our FRET-based probe, **A-1**, could monitor the progression of Zn(ii)–Aβ aggregation, distinct from metal-free Aβ aggregation.

### Screening inhibitors against Zn(ii)–Aβ interaction

**Fig. 4:**
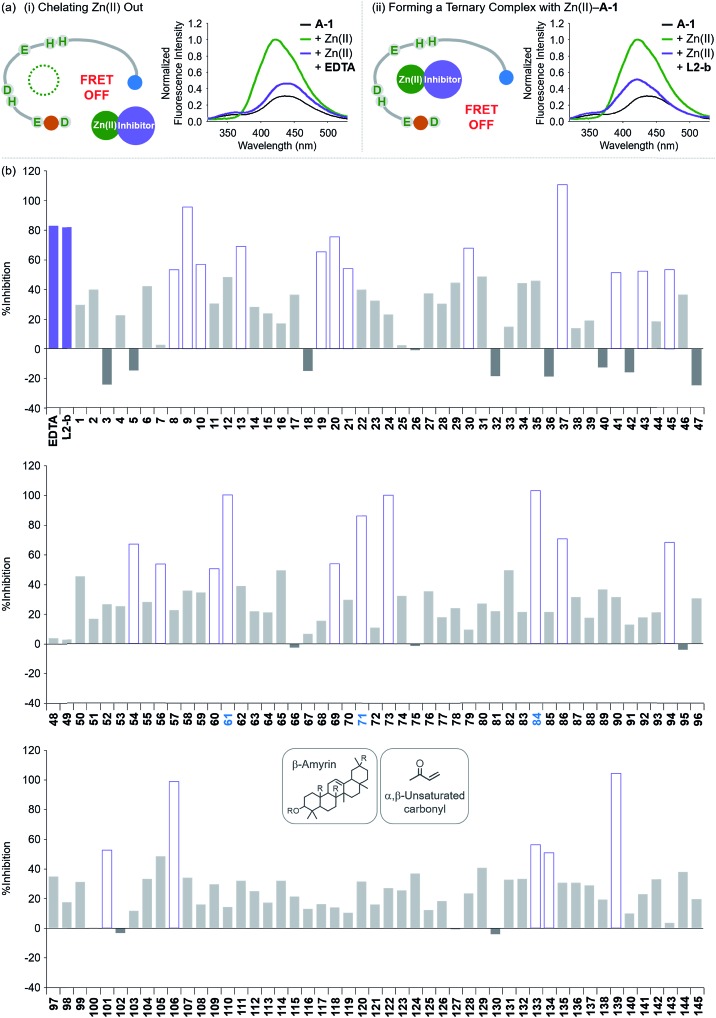
Change in the FRET signal of Zn(ii)-bound **A-1** upon treatment with inhibitors against Zn(ii)–Aβ interaction. (a) Fluorescent responses of **A-1** in the presence of both Zn(ii) and compounds: [(i) **EDTA** and (ii) **L2-b]**. (b) Inhibition (%) of Zn(ii)–**A-1** interaction by incubation with the natural products. Full data sets regarding the inhibition (%) of 145 natural products are summarized in Table S1.† **61**, **71**, and **84** that contain both β-amyrin and α,β-unsaturated carbonyl groups and show >80% inhibition against Zn(ii)–**A-1** interaction are labeled in blue. Conditions: [**A-1**] = 0.3 μM; [ZnCl_2_] = 100 μM; [inhibitor] = 100 μM; incubation for 10 min; room temperature; *λ*_ex_ = 280 nm; *λ*_em_ = 420 nm.

To evaluate whether Zn(ii)-bound **A-1** is an effective identification tool for inhibitors against Zn(ii)–Aβ interaction, alteration of the FRET signal of Zn(ii)–**A-1** was monitored upon addition of the metal chelator (*i.e.*, **EDTA**) or the molecule capable of forming a ternary complex with Zn(ii)–Aβ (*i.e.*, **L2-b**) (Fig. 4a and Table S1†).^29^–^31^ When **EDTA** was introduced to Zn(ii)–**A-1**, the FRET intensity was reduced by 83%, compared to the FRET signal of Zn(ii)–**A-1**, and the emission spectrum was red shifted back to that observed under Zn(ii)-free conditions (Fig. 4a, i). This suggests that Zn(ii) was chelated out from **A-1** by **EDTA**, causing the probe to be unfolded. Furthermore, the treatment of **L2-b** to Zn(ii)–**A-1** exhibited a noticeably weaker FRET signal than Zn(ii)–**A-1** by 82%, but did not present the same emission spectrum as that of Zn(ii)-free **A-1** (Fig. 4a, ii). The fluorescence behavior of **L2-b**-added Zn(ii)–**A-1** implies that a ternary complex [*e.g.*, **L2-b**–Zn(ii)–**A-1**] could be formed and thus Zn(ii) still interacts with **A-1**, but the distance between the FRET donor and acceptor may not be in close proximity. Note that the emission of Trp was not significantly changed at *ca.* 350 nm which was not absorbed by the FRET acceptor upon addition of **EDTA** and **L2-b** (Fig. 4a), indicating that the compounds did not affect the absorption and emission of the FRET donor. Together, our probe, **A-1**, demonstrates the ability to identify molecules with inhibitory activity towards metal–Aβ interaction.

Moving forward, to confirm the screening capability of our FRET-based method to verify molecules as potential inhibitors against metal–Aβ interaction, we built up a chemical library containing 145 natural products that do not absorb the FRET signal of Zn(ii)–**A-1** at *ca.* 420 nm, similar to **EDTA** and **L2-b** (Fig. S6 and Table S1†). The FRET signal of Zn(ii)–**A-1** upon addition of natural products was compared to that of Zn(ii)–**A-1** to calculate % inhibition (Fig. 4b, S7, and Table S1†). In our library, (i) 15 molecules could not affect Zn(ii) binding to **A-1**; (ii) 103 compounds showed 0 to 50% inhibition; (iii) 27 natural products induced a significant decrease in the fluorescence of Zn(ii)–**A-1** by >*ca.* 50% (Fig. 4b and Table S1†). Furthermore, among the 27 natural products (>*ca.* 50% inhibition), 8 compounds (*i.e.*, **9**, **37**, **61**, **71**, **73**, **84**, **106**, and **139**) demonstrated >80% inhibitory activity against Zn(ii)–**A-1** interaction. Three compounds (*i.e.*, **61**, **71**, and **84** out of 8 potent inhibitors; Fig. 4b and Table S1†) contain both β-amyrin moiety and α,β-unsaturated carbonyl groups, previously reported for controlling metal–Aβ aggregation.^43^ Note that the compounds containing an α,β-unsaturated carbonyl moiety could form a covalent adduct with Aβ possibly by reacting with Lys or His.^44^,^45^ To verify the covalent bond formation between one of the effective inhibitors, **61**, and an Aβ fragment (Aβ_28_), the sample containing **61** and Aβ_28_ was monitored by MS. The MS measurement presented a covalent Aβ_28_–**61** adduct at 1244 *m*/*z* (blue peak; Fig. S8a†). In addition, the tandem MS analysis of the peak at 1244 *m*/*z* indicated Aβ_28_ (at 1088 *m*/*z*) and **61** (at 471 *m*/*z*) confirming the formation of the covalent Aβ_28_–**61** adduct (Fig. S8b†). Thus, our inhibitors containing an α,β-unsaturated carbonyl moiety have the potential to bind **A-1**. Overall, inhibitors against Zn(ii)–Aβ interaction could be screened and identified by our probe, **A-1**, showing a variation in its FRET signal in the presence of Zn(ii).

### Influence of inhibitors on toxicity associated with Zn(ii) and Zn(ii)–Aβ

**Fig. 5:**
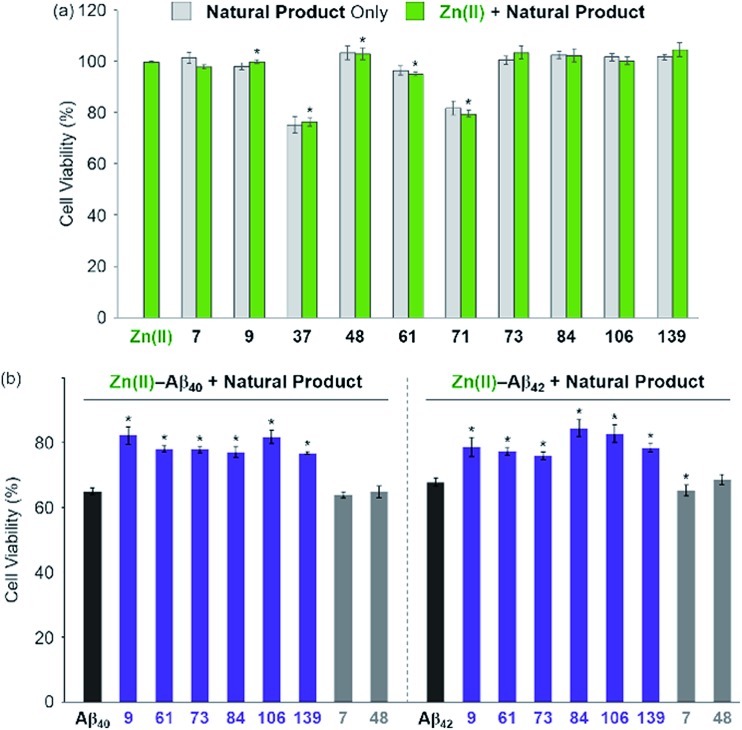
Effect of the selected natural products on the cytotoxicity triggered by Zn(ii) and Zn(ii)–Aβ. (a) Toxicity of the selected natural products with and without Zn(ii) in 5Y cells. Cells were treated with compounds (10 μM) in the absence (light gray) and presence (light green) of Zn(ii) (same equivalent to compounds; 10 μM) for 24 h at 37 °C. (b) Aβ_40_ (left) or Aβ_42_ (right; 10 μM) with Zn(ii) (10 μM) was pre-incubated at room temperature for 1 h and then treated to 5Y cells with compounds (10 μM) for 24 h. Cell viability (%) was determined by the MTT assay compared to that obtained upon treatement with a volume of H_2_O (1% v/v DMSO) equal to the samples added. Error bars represent the standard error of the mean from three independent experiments. *P < 0.05.

The effect of the 8 natural products that showed >80% inhibition against Zn(ii)–**A-1** interaction on the toxicity triggered by metal-free and Zn(ii)-treated Aβ_40_ and Aβ_42_ (two major isoforms of Aβ)^6^,^10^ was determined in living cells. We first examined the toxicity of 10 natural products (*i.e.*, 8 effective natural products: **9**, **37**, **61**, **71**, **73**, **84**, **106**, and **139**; 2 compounds which may not be able to disrupt Zn(ii)–**A-1** interaction: **7** and **48**) in human neuroblastoma SH-SY5Y (5Y) cells. The tested compounds, except for **37** and **71**, were not relatively toxic (>*ca.* 80% of cell viability at more than 10 μM) in the absence and presence of Zn(ii) (Fig. 5a and S9†). Employing the relatively less toxic natural products (*i.e.*, **7**, **9**, **48**, **61**, **73**, **84**, **106**, and **139**) with and without Zn(ii), their impact on the toxicity induced by pre-incubated Aβ_40_ and Aβ_42_ with and without Zn(ii) for 1 h at room temperature was analyzed. The natural products could not ameliorate the toxicity induced by metal-free Aβ (Fig. S10†). On the other hand, as depicted in Fig. 5b (purple), cell survival was improved by 6 natural products, determined as effective inhibitors against metal–**A-1** interaction, even with the species of Zn(ii)–Aβ. As expected, the compounds, **7** and **48**, shown to hinder Zn(ii) binding to Aβ by less than *ca.* 5% (Fig. 4b and Table S1†), were not able to mitigate the toxicity induced by both metal-free and Zn(ii)-associated Aβ (Fig. 5b and S10;† gray). Thus, our FRET-based method employing **A-1** demonstrates its practical utility to determine molecules that can affect metal–Aβ interaction and, as a result, alleviate metal–Aβ-linked cytotoxicity.

## Conclusions

Since metal ions and amyloidogenic peptides (*e.g.*, Aβ) can interact with each other and induce neurotoxicity, our understanding of such complexation is important to reveal their effects in the pathogenesis of neurodegenerative diseases. In order to verify the feasibility of monitoring metal–amyloidogenic peptide interactions, we employed Aβ as an example of amyloidogenic peptides to develop a FRET-based probe, **A-1**, to detect the metal binding of Aβ and the progression of metal–Aβ aggregation effectively and efficiently. Upon addition of Zn(ii), the FRET signal of **A-1** was significantly increased due to the folding of our probe. In addition, when the probe aggregated with Zn(ii), its fluorescent response was altered in a distinct manner from that of metal-free case. Furthermore, by utilizing our FRET-based probe to screen a chemical library (total 145 compounds), we identified 6 natural products capable of significantly modulating metal–Aβ interaction (>80% inhibition) *in vitro* and diminishing cytotoxicity associated with Zn(ii)–Aβ in living cells. Our overall studies illustrate the development of a strategy to monitor metal–Aβ interaction and its applicability towards searching potent inhibitors against metal–Aβ interaction. In the near future, for biological applications, new and optimized probes will be developed to monitor the interaction between Aβ and Zn(ii) or other metal ions, including Cu(ii), showing more sensitive fluorescent responses with lower energy profiles for excitation and emission (*e.g.*, near-infrared region). Applying our tactic to other amyloidogenic peptides, their interactions with metal ions could be, and the inhibitors against metal–amyloidogenic peptide interaction could be identified.

## Conflicts of interest

There are no conflicts to declare.

## Supplementary Material

Supplementary information
