# Correction: Monitoring metal–amyloid-β complexation by a FRET-based probe: design, detection, and inhibitor screening

**DOI:** 10.1039/c9sc90118c

**Published:** 2019-06-17

**Authors:** Hyuck Jin Lee, Young Geun Lee, Juhye Kang, Seung Hyun Yang, Ju Hwan Kim, Amar B. T. Ghisaidoobe, Hyo Jin Kang, Sang-Rae Lee, Mi Hee Lim, Sang J. Chung

**Affiliations:** a Department of Chemistry , Korea Advanced Institute of Science and Technology (KAIST) , Daejeon 34141 , Republic of Korea . Email: miheelim@kaist.ac.kr; b Department of Chemistry , Dongguk University , Seoul 04620 , Republic of Korea . Email: jin0305@dongguk.edu; c Department of Chemistry , Ulsan National Institute of Science and Technology (UNIST) , Ulsan 44919 , Republic of Korea; d School of Pharmacy , Sungkyunkwan University , Suwon 16419 , Republic of Korea . Email: sjchung@skku.edu; e National Primate Research Center (NPRC) , Korea Research Institute of Bioscience and Biotechnology , Cheongju , Chungbuk 28116 , Republic of Korea . Email: srlee@kribb.re.kr

## Abstract

Correction for ‘Monitoring metal–amyloid-β complexation by a FRET-based probe: design, detection, and inhibitor screening’ by Hyuck Jin Lee *et al.*, *Chem. Sci.*, 2019, **10**, 1000–1007.



## 

In their original Edge article, the authors reported a FRET-based probe, **A-1**, composed of amyloid-β_1-21_ (Aβ_1-21_) with a pair of FRET donor and acceptor and used this for monitoring Zn(ii)–Aβ complexation with a turn-on FRET signal and identifying inhibitors against Zn(ii)–Aβ interaction. The authors provided the methods and procedures for experiments, however, more detailed experimental information is provided here to improve clarity for the readers. In this Correction, the authors have provided information about selecting the concentrations of the probe and Zn(ii) and buffer, and detecting a change in FRET depending on the length (sequence) of the probe, along with more discussion on the effects of inhibitors on the FRET of Zn(ii)-added **A-1** and the purpose of designing the probe.

The additional information and references are provided below. The Royal Society of Chemistry apologises for these errors and any consequent inconvenience to authors and readers.

## Results and discussion

### Selection of experimental conditions for FRET measurements

To noticeably monitor the FRET signal of **A-1** upon Zn(ii) binding, the conditions for (i) less and slower aggregation of **A-1** and (ii) its unfolding state prior to treatment with Zn(ii) are required. Thus, a range of nM concentrations of **A-1** were applied to measure its FRET signal. Based on Zn(ii) titration experiments, the concentration of Zn(ii) for fluorescence measurements was chosen (Fig. S3 in the original article). The fluorescence intensity of **A-1** (500 nM) at 420 nm was enhanced upon titration of Zn(ii) and saturated at *ca.* 100 μM Zn(ii). Taken together, a range of nM concentrations of **A-1** (250–500 nM) and 100 μM Zn(ii) were used for our experiments. Note that if 5 μM **A-1** was used for the experiments, we observed that *ca.* 10 μM Zn(ii) (2 equiv. of **A-1**) was required to give an maximum increase in its FRET signal (Fig. S3 in the original article), however, the probe aggregates faster at this concentration, relative to at a nM concentration. In addition, based on the binding affinity of Zn(ii) to full length Aβ_40_ or Aβ_42_ (*ca.* nM to μM depending on the experimental conditions),^1^ our titration data obtained using 500 nM or 5 μM **A-1** are expected. Moreover, the solvent could influence the folding state and FRET response of **A-1**. Therefore, four criteria were considered to select a suitable buffer for our measurements: (i) the solubility of **A-1**, (ii) the aggregation rate of **A-1**, (iii) no other divalent metal ions being present, and (iv) a significant change in the fluorescence of **A-1** by Zn(ii) binding. Following these criteria to select the best solution for our experiments, we examined the change in the FRET signal of **A-1** upon treatment of Zn(ii) in three different solvents; (i) ddH_2_O, (ii) 100 mM Tris (pH 7.0), and (iii) 10% Dulbecco’s Phosphate-Buffered Saline (DPBS that does not contain other divalent metal ions; pH 7.3) (Fig. 1). When **A-1** was in ddH_2_O or Tris buffer, the FRET signal of **A-1** with Zn(ii) was not significantly changed compared to that under Zn(ii)-free conditions. The FRET signal of **A-1**, however, increased by >*ca.* 2-fold upon treatment with Zn(ii) in 10% DPBS. Using our criteria, 10% DPBS (the buffer solution without other divalent metal ions) was chosen for our experiments. Note that we cannot rule out the possibility of forming Zn(ii)–buffer (phosphate) complexes and a ternary complex of Zn(ii), buffer, and **A-1**, similar to previously reported studies,^2^,^3^ which may affect the FRET signal of **A-1**.

**Fig. 1:**
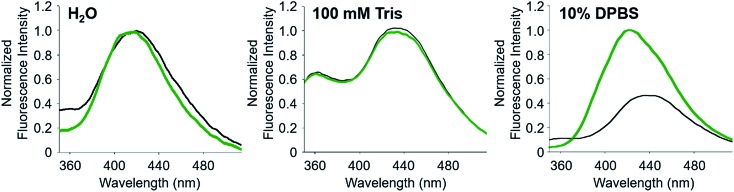
FRET signals of **A-1** with addition of Zn(ii) under various conditions. Emission spectra of **A-1** with (green) and without (black) Zn(ii) in ddH_2_O (left), 100 mM Tris (middle), and 10% DPBS (right). Conditions: [**A-1**] = 500 nM; [ZnCl_2_] = 100 μM; *λ*_ex_ = 280 nm; room temperature.

### Change in FRET depending on the length of FRET model peptides

Since FRET occurs through the long-range dipole–dipole interaction between donors and acceptors, it is sensitive to the orientation and distance of the FRET pair.^4^,^5^ When the FRET of the model peptides with different lengths was monitored, Aβ_1-21_ (**A-1**) only showed a strong increase in FRET upon Zn(ii) addition, but Aβ_1-17_ and Aβ_1-18_ indicated a very small increase in FRET in the presence of Zn(ii) in 10% DPBS (Fig. 2a). In the case of Aβ_1-22_, the probe showed a decrease in fluorescence upon Zn(ii) addition. In addition, Aβ_1-19_ exhibited no FRET signal but presented a noticeable intensity in fluorescence at *ca.* 370 nm corresponding to Trp fluorescence. Note that the FRET signal of the model peptides was observed in 10% DPBS; however, it was not shown in 10% PBS (Fig. 2). These data support that **A-1** was the model peptide suitable for observing FRET upon Zn(ii)-mediated conformational change in 10% DPBS.

**Fig. 2:**
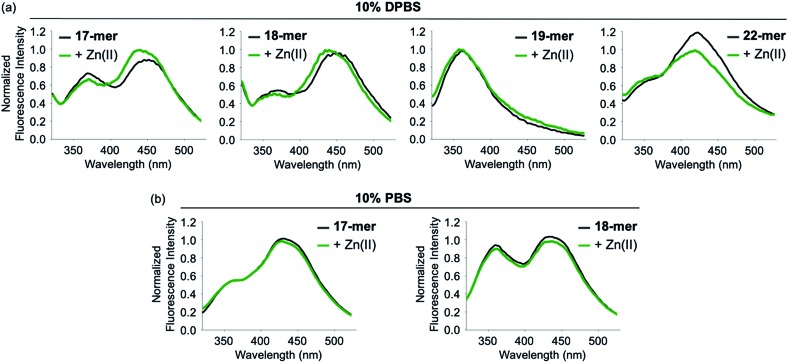
Change in Zn(ii)-dependent fluorescence with the FRET model peptides (Aβ_1-17_, Aβ_1-18_, Aβ_1-19_, and Aβ_1-22_). (a) Fluorescent responses of Aβ_1-17_, Aβ_1-18_, Aβ_1-19_, and Aβ_1-22_ to Zn(ii) in 10% DPBS. (b) Fluorescence behavior of Aβ_1-17_ and Aβ_1-18_ with and without Zn(ii) in 10% PBS. Conditions: [FRET model peptide] = 500 nM; [Zn(ii)] = 100 μM.

### Variation of the FRET signal of Zn(ii)-added **A-1** upon incubation with a chelator

Considering the general properties of fluorophores, the change in *λ*_max_ upon Zn(ii) coordination to **A-1** suggests that the microenvironment of the fluorescent acceptor probably becomes hydrophobic. The addition of metal chelator, **L2-b** (structure shown in Table S1 in the original article), changed the fluorescence intensity of the fluorescent acceptor; however, it did not vary *λ*_max_ of the emission. This implies that the microenvironment of the fluorescent acceptor does not significantly change, and Zn(ii) is still coordinated to **A-1**. Whereas, the treatment of a well-known metal chelator, EDTA (structure shown in Table S1 in the original article), altered *λ*_max_ of the emission, which became the same as that of the peptide itself with a decrease in the fluorescence intensity, suggesting that the microenvironment of the fluorescent acceptor was similar to that of **A-1** itself showing no significant FRET. Since EDTA is a strong metal chelator, it could extract Zn(ii) from **A-1**. Moreover, since **A-1** aggregates with and without Zn(ii), a heterogeneous population of its conformations upon incubation in solution may be another factor that influences the chelators’ interaction towards the Zn(ii)-added probe and affects the FRET signal. Together, these properties may explain the increased fluorescence intensity compared to that of **A-1**.

### The purpose of designing **A-1**

**A-1** was designed as an *in vitro* probe capable of Zn(ii) binding showing FRET. This probe is composed of the metal binding site in Aβ; thus, it can be a model for Zn(ii)–Aβ upon Zn(ii) binding, and can be used for identifying potential inhibitors against Zn(ii)–Aβ interaction in an efficient and effective manner. The inhibitors determined by Zn(ii)–**A-1** based on FRET could be further evaluated in biological systems to confirm their biological use as inhibitors against Zn(ii)–Aβ complexes.
