# Supplementary material for: Monitoring metal–amyloid-β complexation by a FRET-based probe: design, detection, and inhibitor screening
Source: Chem Sci. 2018 Dec 6;10(4):1000–7. doi: 10.1039/c8sc04943b (PMC6349019; doi:10.1039/c8sc04943b)
Supplement: Supplementary file 1 [file SC-010-C8SC04943B-s001.pdf]

Electronic Supplementary Material (ESI) for Chemical Science.
This journal is © The Royal Society of Chemistry 2019

Supplementary Information

for

Monitoring metal-amyloid-β complexation by a FRET-based probe: design, detection, and inhibitor screening

Hyuck Jin Lee,[‡a] Young Geun Lee,[‡b] Juhye Kang,[‡ac] Seung Hyun Yang,[b]
Ju Hwan Kim,[d] Amar B.T. Ghisaidoobe,[b] Hyo Jin Kang,*[b] Sang-Rae Lee,*[e]
Mi Hee Lim,*[a] and Sang J. Chung*[bd]

[a]Department of Chemistry, Korea Advanced Institute of Science and Technology (KAIST), Daejeon 34141, Republic of Korea

[b]Department of Chemistry, Dongguk University, Seoul 04620, Republic of Korea

[c]Department of Chemistry, Ulsan National Institute of Science and Technology (UNIST), Ulsan 44919, Republic of Korea

[d]School of Pharmacy, Sungkyunkwan University, Suwon 16419, Republic of Korea

[e]National Primate Research Center (NPRC), Korea Research Institute of Bioscience and Biotechnology, Cheongju, Chungbuk 28116, Republic of Korea

[‡]These authors contributed equally to the work.

*To whom correspondence should be addressed: sjchung@skku.edu (S.J.C.); miheelim@kaist.ac.kr (M.H.L.); srlee@kribb.re.kr (S.-R.L.); jin0305@dongguk.edu (H.J.K.)

Table of Contents

**Experimental Section**		S3
**References**		S10
**Table S1**	Chemical structures of **EDTA**, **L2-b**, and the natural products in our chemical library and their inhibition values (%)	S12
**Fig. S1**	Fluorescent response of **A-1** upon treatment with Zn(II) or Cu(II)	S21
**Fig. S2**	Zn(II) binding of **A-1**, monitored by MS	S22
**Fig. S3**	Variation of the FRET intensity of **A-1** (at 420 nm) upon titration with Zn(II)	S23
**Fig. S4**	Change in the FRET signal of **A-1** following the incubation time without Zn(II)	S24
**Fig. S5**	Time-dependent aggregation progression of $A\beta_{40}$ with and without Zn(II)	S25
**Fig. S6**	Absorption spectra of **EDTA**, **L2-b**, and the selected natural products in the absence and presence of Zn(II)	S26
**Fig. S7**	FRET responses of **A-1** to Zn(II) without and with the selected natural products	S27
**Fig. S8**	Mass spectrometric analysis of the sample containing $A\beta_{28}$ with the natural product, **61**	S28
**Fig. S9**	Toxicity of the selected natural products with and without Zn(II) in 5Y cells	S29
**Fig. S10**	Effect of the selected natural products on the toxicity triggered by metal-free Aβ in 5Y cells	S30

Experimental Section

Materials and Methods

All chemical reagents (reagent grade) were purchased from Sigma Aldrich or Tokyo Chemical Industry (Tokyo, Japan) and used as received. Fmoc-protected amino acids were purchased from Chemimpex (Wood Dale, IL, USA) and GL biochem (Shanghai, China). Full length Aβ (*i.e.*, $Aβ_{40}$ and $Aβ_{42}$) peptides were purchased from Anaspec (Fremont, CA, USA) ($Aβ_{40}$ = DAEFRH-DSGYEVHHQKLVFFAEDVGSNKGAIIGLMVGGVV; $Aβ_{42}$ = DAEFRHDSGYEVHHQKLV-FFAEDVGSNKGAIIGLMVGGVVIA). Reaction products were analyzed using high performance liquid chromatography (HPLC), matrix associated laser desorption ionization time-of-flight mass spectrometry (MALDI-TOF MS), and nuclear magnetic resonance spectroscopy (NMR). HPLC analysis was performed using the Waters photodiode array detector 996, separation module 2695, and XBridgeTM C18 column (5 µm, 4.6 x 250 mm). Peptide purification was performed by the Waters XBridgeTM Prep C18 column (5 µm, 19 x 250 mm). Purified peptide samples and their Zn(II) binding studies were analyzed by a Bruker MALDI-TOF mass spectrometer (Sogang University, Seoul, Republic of Korea) and a Q Exactive Plus Orbitrap mass spectrometer (Thermo Fisher Scientific, Waltham, MA, USA), respectively. Fluorescence was recorded on a PerkinElmer LS55 fluorescence spectrometer (PerkinElmer, Waltham, MA, USA) and HORIBA PTI QuantaMaster 8000 fluorescence spectrometer (HORIBA, Kyoto, Japan). TEM images were taken by a JEOL JEM-2100 transmission electron microscope [UNIST Central Research Facilities (UCRF), Ulsan, Republic of Korea] and a Tecnai F30 (FEI) transmission electron microscope [KAIST Analysis Center for Research Advancement (KARA), Daejeon, Republic of Korea]. Absorbance values of compounds and the MTT assay and the fluorescence intensity of ThT were measured using a Molecular Devices

SpectraMax M5e microplate reader (Sunnyvale, CA, USA). The complex formation between Aβ fragments and the natural product was analyzed by the MicrOTOF-QII Hybrid Qquadrupole-Time of Flight mass spectrometer (Bruker, Billercia, Massachusetts, USA) equipped with an ESI source [KARA, KAIST, Republic of Korea]

Synthesis and Purification of A-1. A-1 was synthesized through solid phase peptide synthesis (SPPS) using the 2-chlorotritylchloride resin (loading = 1.2 mmol/1 g resin). The resin was swelled with dry dichloromethane (DCM) for 30 min at room temperature. Immobilization of the Fmoc-amino acid was carried out on 1 g resin using 2 equiv of the Fmoc amino acid (2.4 mmol) and 4 equiv of *N*,*N*-diisopropylethylenediamine (DIPEA) for 1 h at room temperature. Fmoc de-blocking was achieved twice for 10 min at room temperature using 20% (v/v) piperidine in *N*,*N*-dimethylformamide (DMF) followed by washing with DMF. Peptide coupling reactions were executed according to a pre-activation (10 min) protocol using 4 equiv of the appropriate amino acid, 4 equiv of 2-(1*H*-benzotriazol-1-yl)-1,1,3,3-tetramethyluronium hexafluorophosphate (HBTU), 4 equiv of 1-hydroxy-benzotriazole (HOBt), and 8 equiv of DIPEA. Then, the pre-activated solution was added to the resin and allowed to shake for 2 h at 30 °C. Using the above protocol, the 21-mer peptide, **A-1** (DDnapEFRHDSGYEVHHQKLVFFW), composed of natural amino acids and/or a side chain-modified aspartic acid with 1-naphthylethylenediamine (Dnap) was synthesized. Cleavage of the final peptide from the resin and deprotection of the acid-labile protective groups were accomplished using a mixture of trifluoroacetic acid (TFA), thioanisole, ethanedithiol, and double-distilled water (ddH_2O) (94/1/2.5/2.5, v/v/v/v). The crude peptide was precipitated from the cleavage solution and thoroughly washed with ice-cold diethyl ether (Et_2O). After preparative HPLC purification using a gradient of acetonitrile (CH_3CN) with 0.075% TFA

in ddH_2O containing 0.1% TFA, the product fractions were pooled and lyophilized to give a white powder. The identity of **A-1** was confirmed using MALDI-TOF MS (Calcd, 2860.342; found, 2860.206).

Side Chain-Functionalized Fmoc-Asp(Nap)-OH (Fmoc-Dnap). Fmoc-Asp(Nap)-O*t*Bu: Orthogonal protected Fmoc-Asp-O*t*Bu (2.0 g, 4.9 mmol) was dissolved in DCM (0.2 M), and subsequently 1-hydroxy-7-azabenzotriazole (HOAt, 1 equiv, 0.65 g, 4.9 mmol), 1-ethyl-3-(3-dimethylaminopropyl)carbodiimide (EDC, 2 equiv, 1.9 g, 9.7 mmol), and DIPEA (3 equiv, 2.5 mL, 15 mmol) were added. The reaction mixture was stirred for 10 min at 0 °C followed by the addition of *N*-naphthylethylenediamine (Nap; 2 equiv, 2.5 g, 9.7 mmol) and DIPEA (2 equiv, 1.7 mL, 9.7 mmol). The reaction mixture was stirred for 20 min at 0 °C and stirring was continued at room temperature for additional 4 h. After complete disappearance of the starting material was confirmed by thin layer chromatography (TLC), the reaction mixture was diluted with DCM and subsequently washed with 1 M HCl (aq, 3x), saturated $NaHCO_3$ (aq, 3x), and brine (3x), dried Na_2SO_4, filtered, and concentrated *in vacuo*. The residue was purified using silica gel column chromatography [hexanes/ethyl acetate (EtOAc) = 2/1 → 1/1) to give the titled compound as a greenish solid (2.2 g; yield = 79%). ^{1}H NMR [500 MHz, $CDCl_3$, δ (ppm)]: 7.78 (s, 1H), 7.67 (d, J = 7.3 Hz, 3H), 7.46 (d, J = 7.4 Hz, 2H), 7.35 (s, 2H), 7.31 (t, J = 7.5 Hz, 2H), 7.23 (dd, J = 14.3, 7.2 Hz, 2H), 7.14 (d, J = 8.1 Hz, 1H), 6.47 (d, J = 7.5 Hz, 1H), 6.13 (s, 1H), 5.94 (d, J = 7.4 Hz, 1H), 5.22 (s, 1H), 4.41 (s, 1H), 4.31-4.22 (m, 1H), 4.21-4.14 (m, 1H), 4.06 (t, J = 7.0 Hz, 1H), 3.58 (d, J = 4.1 Hz, 2H), 3.33 (s, 2H), 2.85-2.74 (m, 1H), 2.70 (dd, J = 15.4, 2.5 Hz, 1H), 1.39 (s, 9H). ^{13}C NMR [125 MHz, $CDCl_3$, δ (ppm)]: 171.1, 170.0, 156.2, 143.8, 143.2, 141.3, 134.3, 128.6, 127.7, 127.1, 126.5, 125.9, 125.2, 124.9, 123.4, 120.3, 120.0, 117.5, 103.8, 82.7, 67.2,

51.5, 47.0, 44.8, 39.0, 38.4, 27.9. HRMS: Calcd, 524.2180; Found, 524.2181.

Fmoc-Asp(Nap)-OH: Fmoc-Asp(Nap)-O*t*Bu (**1**, 1.5 g, 2.6 mmol) was co-evaporated with DCM and dissolved in a solution of 30% TFA in DCM at 0 °C. The reaction mixture was allowed to warm up to room temperature for 2 h, after which the volatiles were removed *in vacuo*. The residue was subjected to silica gel column chromatography (hexanes/EtOAc 1/5 → 1/15) to give the titled compound as a light greenish solid (1.3 g; 93%). ^{1}H NMR [500 MHz, DMSO-d_6, δ (ppm)]: 12.73 (s, 7H), 8.18 (t, J = 5.7 Hz, 35H), 8.08 (d, J = 8.1 Hz, 34H), 7.88 (d, J = 7.5 Hz, 68H), 7.77-7.67 (m, 100H), 7.62 (d, J = 8.3 Hz, 30H), 7.47-7.35 (m, 136H), 7.35-7.23 (m, 103H), 7.10 (d, J = 8.1 Hz, 7H), 6.57 (t, J = 10.7 Hz, 34H), 6.21 (s, 33H), 4.43 (td, J = 8.1, 5.6 Hz, 6H), 4.26 (dd, J = 7.0, 2.6 Hz, 11H), 4.21 (dd, J = 14.3, 7.5 Hz, 8H), 3.46-3.32 (m, 37H), 3.27 (d, J = 3.8 Hz, 15H), 2.67 (d, J = 5.4 Hz, 3H), 2.64 (d, J = 5.3 Hz, 4H), 2.61-2.51 (m, 7H), 1.40-1.13 (m, 5H). ^{13}C NMR [125 MHz, DMSO-d_6, δ (ppm)]: 173.6, 170.2, 156.3, 144.3, 144.3, 144.3, 141.2, 134.5, 128.4, 128.1, 127.6, 127.3, 126.1, 125.7, 124.5, 123.4, 121.9, 120.6, 116.0, 103.2, 66.1, 51.2, 47.1, 43.4, 38.2, 37.6. HRMS: Calcd, 580.2806; Found, 580.2811.

Preparation of the Samples Containing A-1. A-1 was dissolved in hexafluoroisopropanol (HFIP, 1 mM) and allowed to stand for 1 h at room temperature. The resultant solution of **A-1** was aliquoted, lyophilized, and stored at -20 °C. The obtained powder was dissolved in a mixture of DMSO/ddH_2O (1:1, v/v) to give the final concentration of 1 mM. To aid the solvation of the peptide, the mixture was vortexed for 1 min followed by sonication for 10 min at room temperature. The resultant solution was allowed to be at room temperature for specified incubation time points. During fluorescence experiments, the solution of **A-1** was stored at 4 °C.

Zn(II) Binding Studies of A-1. The solution of **A-1** (20 μM) and $ZnCl_2$ (2.0 mM) was prepared in 20 mM ammonium acetate buffer (pH 7.4) and incubated for 1 h without agitation at room temperature. The MS analysis was performed using the Q Exactive Plus Orbitrap mass spectrometer (Thermo Fisher Scientific) with an electrospray ionization (ESI) source.

Modeling of A-1 with and without Zn(II). The structures of metal-free **A-1** and Zn(II)-bound **A-1** were generated with modifications of the previously reported structures of metal-free Aβ (PDB: 1AMC)[1] and Zn(II)-bound Aβ (PDB: 1ZE9)[2] by Discovery Studio Visualizer.

Fluorescent Measurements. **A-1** (250-500 nM) and $ZnCl_2$ (100 μM) with and without compounds (*i.e.*, **L2-b**, **EDTA**, natural products; 100 μM) were mixed in 10% DPBS for 10 min without agitation. The mixture was excited at 280 nm. The FRET signal of Zn(II)-treated **A-1** was recorded from 300 to 600 nm. Particularly, the emission of **A-1** at 420 nm showed > *ca.* 2-fold increase in the presence of $ZnCl_2$, relative to that of **A-1** itself. The buffer for our measurements was selected based on (i) the solubility and (ii) aggregation rate of **A-1** as well as (iii) no presence of other divalent metal ions. In addition, based on Zn(II) titration experiments, the concentration of Zn(II) for fluorescence measurements was chosen. The fluorescence intensity of **A-1** (500 nM) at 420 nm was enhanced upon titration with Zn(II) and was saturated at *ca*. 100 μM of Zn(II). Thus, 500 nM of **A-1** and 100 μM of Zn(II) were used for fluorescence measurements. Moreover, Zn(II) titration to **A-1** (5 μM) was conducted in order to determine the dissociation constant (K_d) of Zn(II)–**A-1**. The K_d value was determined following previously reported methods.[3-6] Furthermore, the fluorescent response of **A-1** (500 nM) in the presence of

Cu(II) (1 equiv to **A-1**) was monitored. Upon incubation with Cu(II), the fluorescent intensity of **A-1** was quenched.

Time-dependent Fluorescence Measurements. A-1 (250 nM) was dissolved in 10% DPBS and incubated with and without $ZnCl_2$ (100 μM) for 10 h. Fluorescence was recorded for 10 h with an interval of 5 min at room temperature.

Morphologies of the Aggregates of A-1 and Aβ$_{40}$ with and/or without Zn(II). A-1 (2.5 μM) was incubated with $ZnCl_2$ (1.0 mM) in 10% DPBS at room temperature for 10 h without agitation. In addition, Aβ$_{40}$ (20 μM) was incubated with and without $ZnCl_2$ (20 μM) in a buffered solution (20 mM HEPES, pH 7.4, 150 mM NaCl) at 37 °C for 10 h with constant agitation. The samples for TEM were prepared according to previously reported methods.[7,8] Glow-discharged grids (Formvar/Carbon 300-mesh, Electron Microscopy Sciences, Hatfield, PA, USA) were treated with the samples from different incubation time points [1, 2, 3, 4, 5, 6, 7, 8, 9, and 10 h for Zn(II)-treated **A-1** (10 μL); 0.5, 3, 5, 7, and 10 h for metal-free Aβ$_{40}$ and Zn(II)-added Aβ$_{40}$ (5 μL)] for 2 min at room temperature. Excess sample was removed with filter paper and washed with ddH_2O. Each grid was treated with uranyl acetate (1% w/v ddH_2O; 5 μL) for 1 min. Excess stain was blotted off and the grids were air dried for at least 20 min at room temperature. Images from each sample were taken at 200 kV with 25,000x magnification [UCRF, Ulsan, Republic of Korea (for Zn(II)-treated **A-1** samples)] and 29,000x magnification [KARA, Daejeon Republic of Korea (for the samples of metal-free and Zn(II)-bound Aβ$_{40}$)].

Thioflavin-T (ThT) Assay. The kinetics of the formation of β-sheet-rich Aβ_{40} aggregates were monitored by the ThT assay according to previous reported methods.[9,10] Each Aβ_{40} sample (20 μM) was obtained after different incubation time points (up to 12 h) at 37 °C with constant agitation (in 20 mM HEPES, pH 7.4, 150 mM NaCl) followed by treatment with ThT (20 μM). After 20 min incubation, the fluorescence intensity of ThT (λ_{ex} = 440 nm; λ_{em} = 490 nm) was measured using a microplate reader, and normalized compared to that of 12 h incubated metal-free Aβ_{40} samples.

Absorption Spectra of Inhibitors. The solutions of the inhibitors, *i.e.*, **EDTA**, **L2-b**, **7**, **9**, **48**, **61**, **73**, **84**, **106**, and **139** (100 μM; 2% v/v DMSO), were prepared in 10% DPBS. Absorption spectra of the inhibitors with and without $ZnCl_2$ (100 μM) were obtained by a microplate reader at room temperature.

Analysis of the Covalent Bond Formation between Aβ Fragments and the Inhibitor (61). Aβ_{28} (20 μM) was incubated with **61** (100 μM; 2% v/v DMSO; containing an α,β-unsaturated carbonyl group) in 20 mM ammonium acetate (pH 7.4) for 2 h at room temperature without agitation. The incubated samples were injected into the MicrOTOF-QII Hybrid Quadrupole-Time of Flight mass spectrometer (Bruker, Billercia, Massachusetts, USA) equipped with an ESI source (KARA, KAIST, Republic of Korea).

Cell Studies. The human neuroblastoma SH-SY5Y (5Y) cell line was purchased from the American Type Culture Collection (ATCC, Manassas, VA, USA). The cell line was maintained in media containing 50% minimum essential medium (MEM) and 50% F12 (GIBCO), and

supplemented with 10% fetal bovine serum (Sigma-Aldrich) and 100 U/mL penicillin (GIBCO). Cells were grown and maintained at 37 °C in a humidified atmosphere with 5% CO_2. The cell culture used in this work did not indicate mycoplasma contamination. Cell viability upon treatment with compounds was determined by the MTT assay [MTT = 3-(4,5-dimethylthiazol-2-yl)-2,5-diphenyltetrazolium bromide]. Cells were seeded in a 96 well plate (15,000 cells in 100 μL per well). Cells were treated with pre-incubated samples containing Zn(II) and/or Aβ for 1 h at room temperature without agitation followed by addition of the selected natural products. After 24 h incubation, MTT [25 μL of 5 mg/mL in PBS (pH 7.4, GIBCO)] was added to each well and the plate was incubated for 4 h at 37 °C. Formazan produced by cells was solubilized using an acidic solution of *N*,*N*-dimethylformamide (DMF, pH 4.5, 50% v/v, aq) and sodium dodecyl sulfate (SDS, 20% w/v) overnight at room temperature in the dark. The absorbance was measured at 600 nm by a microplate reader. Cell viability was calculated relative to that of the cells containing a volume of ddH_2O (containing 1% DMSO) equal to the volume of protein sample added.

Statistical Analysis. All data present mean ± standard error of the mean (S.E.M.). For comparisons between two groups, Student's two-tailed unpaired *t* test was employed. Statistical difference was considered significant at $^*P < 0.05$.

References

1 J. Talafous, K. J. Marcinowski, G. Klopman and M. G. Zagorski, *Biochemistry*, 1994, **33**, 7788–7796.

2 S. Zirah, S. A. Kozin, A. K. Mazur, A. Blond, M. Cheminant, I. Ségalas-Milazzo, P. Debey

and S. Rebuffat, *J. Biol. Chem.*, 2006, **281**, 2151–2161.

3 W. Garzon-Rodriguez, A. K. Yatsimirsky and C. G. Glabe, *Bioorg. Med. Chem. Lett.*, 1999, **9**, 2243–2248.

4 V. Tõugu, A. Karafin and P. Palumaa, *J. Neurochem.*, 2008, **104**, 1249–1259.

5 S. L. Leong, T. R. Young, K. J. Barnham, A. G. Wedd, M. G. Hinds, Z. Xiao and R. Cappai, *Metallomics*, 2014, **6**, 105–116.

6 V. Tran, M. C. H. Park and C. Stricker, *Cell Calcium*, 2018, **71**, 86–94.

7 J.-S. Choi, J. J. Braymer, R. P. R. Nanga, A. Ramamoorthy and M. H. Lim, *Proc. Natl. Acad. Sci. U. S. A.*, 2010, **107**, 21990–21995.

8 M. W. Beck, S. B. Oh, R. A. Kerr, H. J. Lee, S. H. Kim, S. Kim, M. Jang, B. T. Ruotolo, J.-Y. Lee and M. H. Lim, *Chem. Sci.*, 2015, **6**, 1879–1886.

9 T. S. Choi, H. J. Lee, J. Y. Han, M. H. Lim and H. I. Kim, *J. Am. Chem. Soc.*, 2017, **139**, 15437–15445.

10 H. J. Lee, M. G. Savelieff, J. Kang, M. B. Brophy, T. G. Nakashige, S. J. C. Lee, E. M. Nolan and M. H. Lim, *Metallomics*, 2018, **10**, 1116–1127.

Table S1 Chemical structures of **EDTA**, **L2-b**, and the natural products in our chemical library and their inhibition values (%) of the interaction between Zn(II) and **A-1**. The inhibition values (%) are presented in parenthesis.

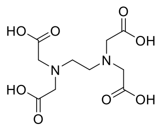	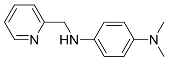		
**EDTA** (83%)	**L2-b** (82%)		
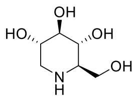	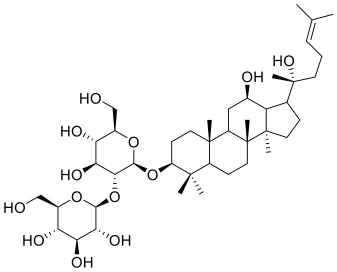		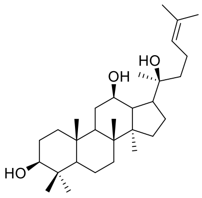
**1** (30%)	**2** (40%)		**3** (-24%)
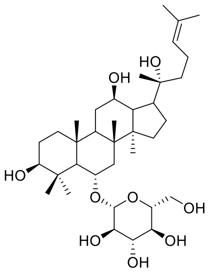	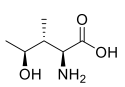	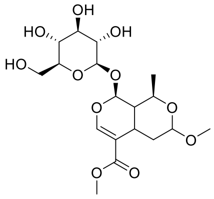	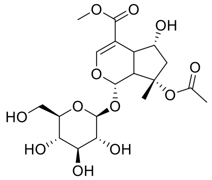
**4** (23%)	**5** (-15%)	**6** (42%)	**7** (2.4%)
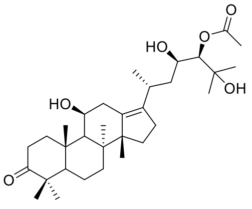	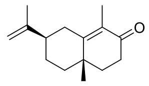	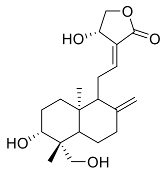	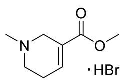
**8** (53%)	**9** (96%)	**10** (57%)	**11** (31%)

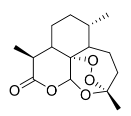	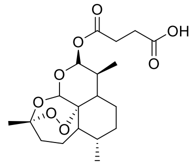	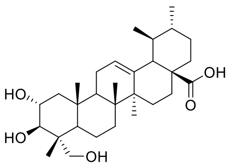	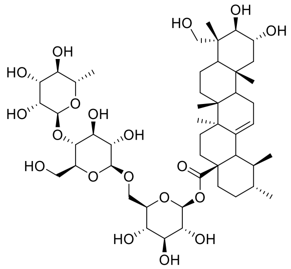
**12** (48%)	**13** (69%)	**14** (28%)	**15** (24%)
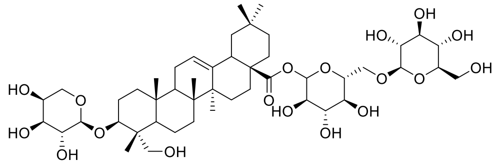		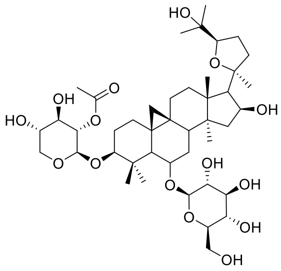	
**16** (17%)		**17** (37%)	
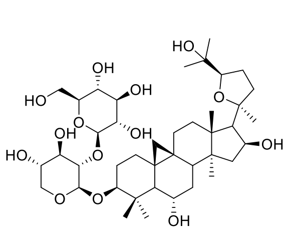		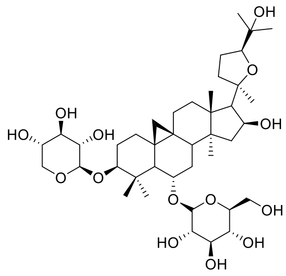	
**18** (-15%)		**19** (66%)	
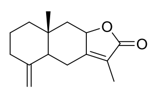	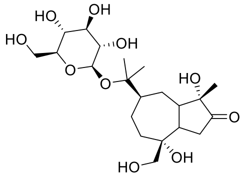	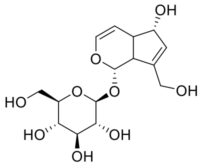	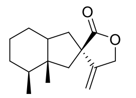
**20** (76%)	**21** (54%)	**22** (40%)	**23** (33%)
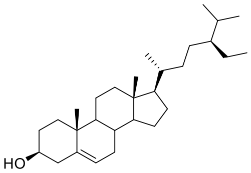		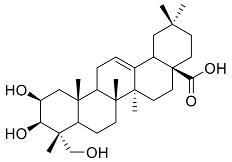	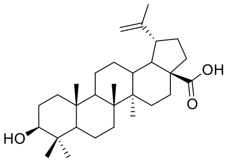
**24** (23%)		**25** (2.4%)	**26** (-1.1%)
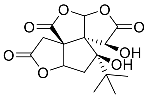	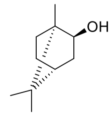	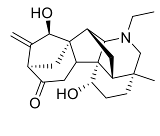	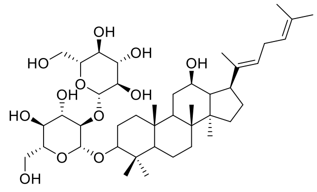
**27** (37%)	**28** (31%)	**29** (44%)	**30** (68%)

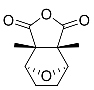	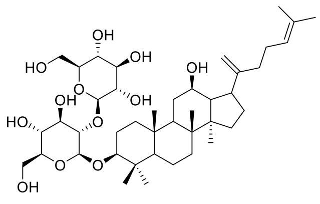	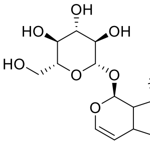	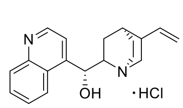
**31** (49%)	**32** (-19%)	**33** (15%)	**34** (44%)
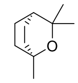	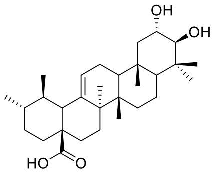	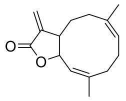	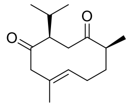
**35** (46%)	**36** (-19%)	**37** (110%)	**38** (14%)
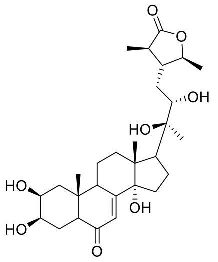	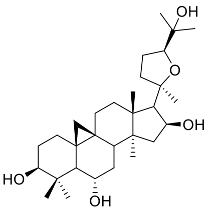	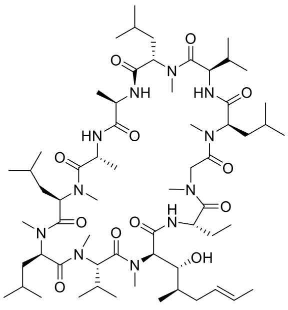	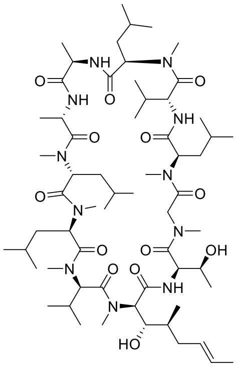
**39** (19%)	**40** (-13%)	**41** (51%)	**42** (-16%)
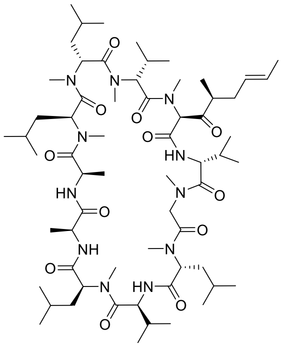	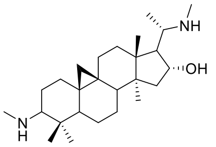	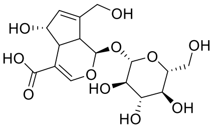	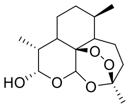
**43** (52%)	**44** (19%)	**45** (53%)	**46** (37%)
	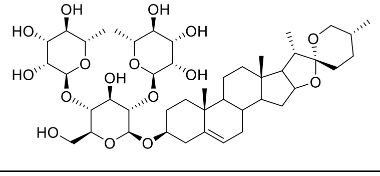		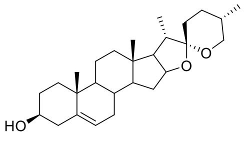
	**47** (-25%)		**48** (3.9%)

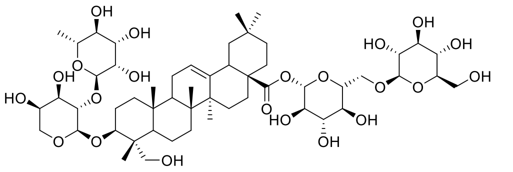		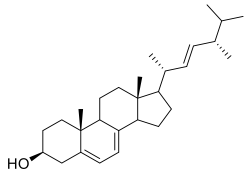	
**49** (2.8%)		**50** (46%)	
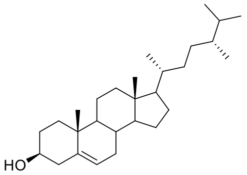	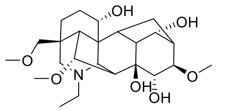	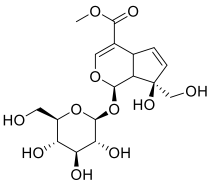	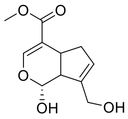
**51** (17%)	**52** (27%)	**53** (25%)	**54** (67%)
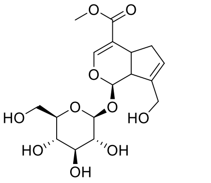	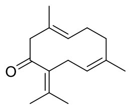	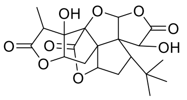	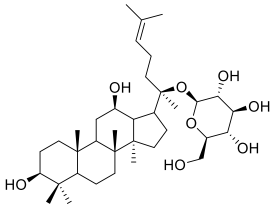
**55** (28%)	**56** (54%)	**57** (23%)	**58** (36%)
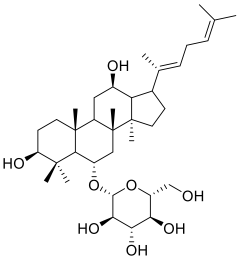		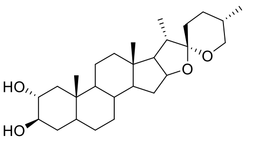	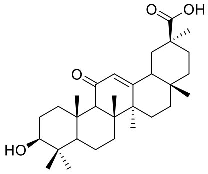
**59** (35%)		**60** (51%)	**61** (100%)
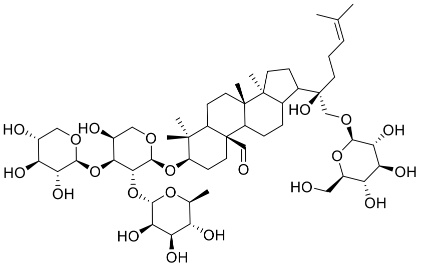		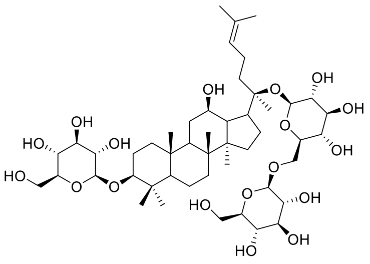	
**62** (39%)		**63** (22%)	
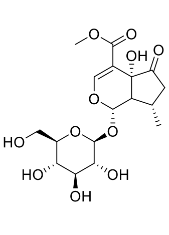	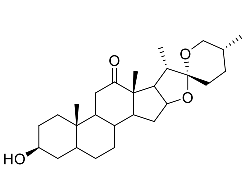	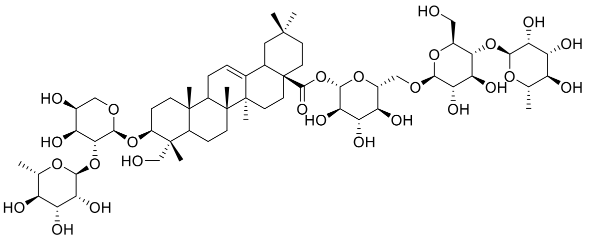	
**64** (22%)	**65** (50%)	**66** (-2.4%)	

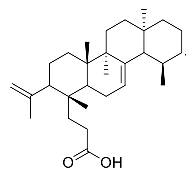	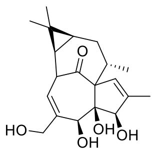	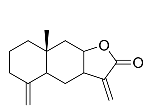	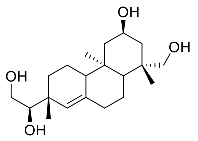
**67** (6.8%)	**68** (16%)	**69** (54%)	**70** (30%)

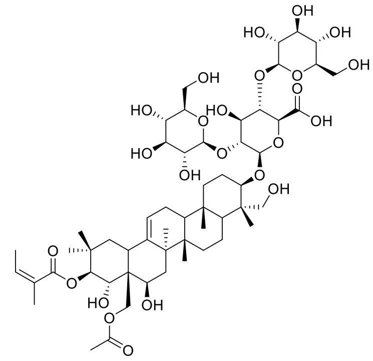	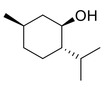	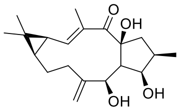	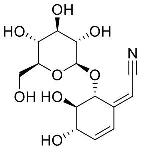
**71** (86%)	**72** (11%)	**73** (100%)	**74** (32%)

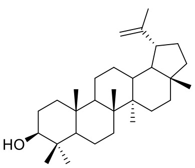	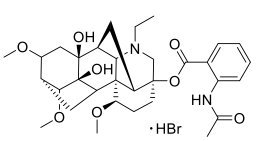	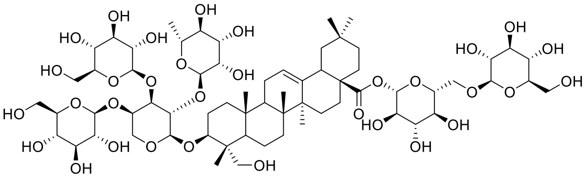
**75** (-1.4%)	**76** (35%)	**77** (18%)

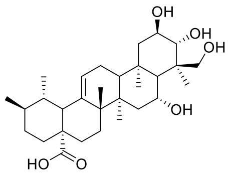	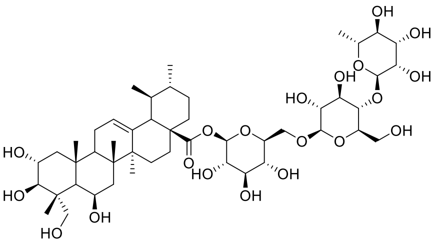	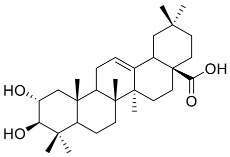
**78** (24%)	**79** (9.6%)	**80** (27%)

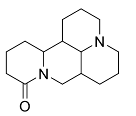	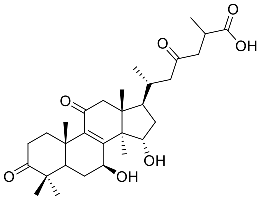	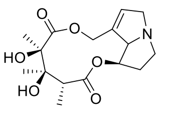
**81** (22%)	**82** (50%)	**83** (21%)

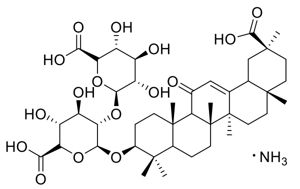

84 (103%)

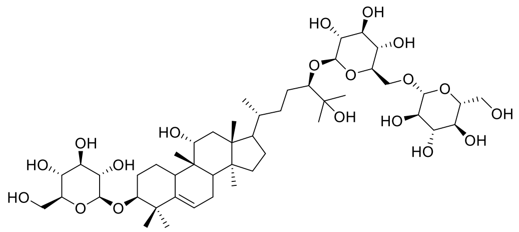

85 (22%)

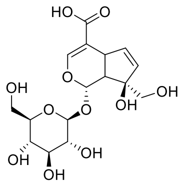

86 (71%)

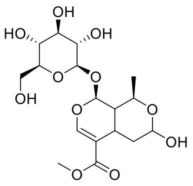

87 (32%)

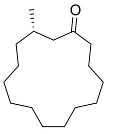

88 (18%)

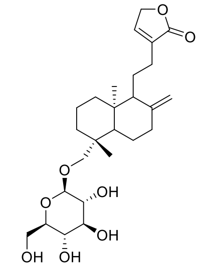

89 (37%)

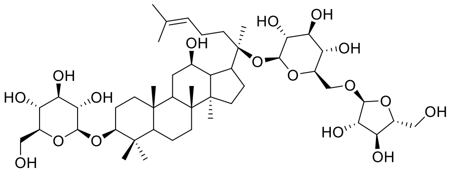

90 (32%)

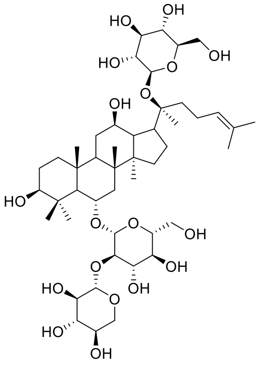

91 (13%)

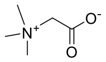

92 (18%)

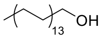

93 (21%)

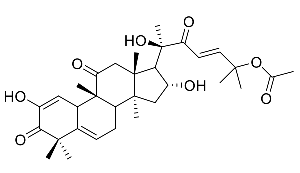

94 (68%)

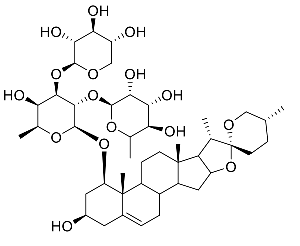

95 (-4.0%)

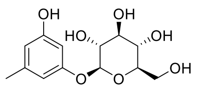

96 (31%)

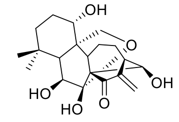

97 (35%)

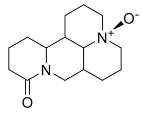

98 (18%)

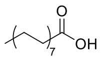

99 (31%)

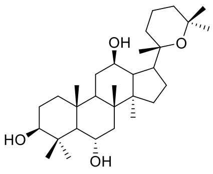

100 (0.30%)

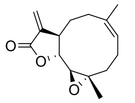

101 (53%)

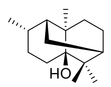

102 (-3.2%)

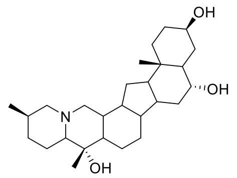

103 (12%)

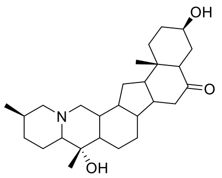

104 (33%)

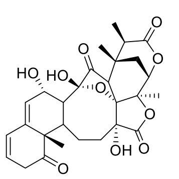

105 (49%)

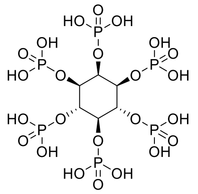

106 (99%)

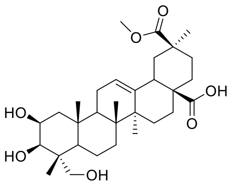

107 (34%)

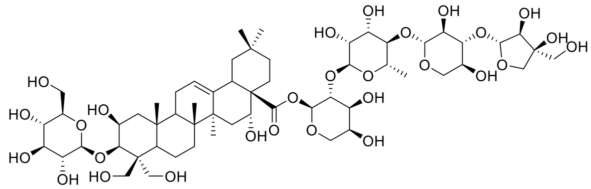

108 (16%)

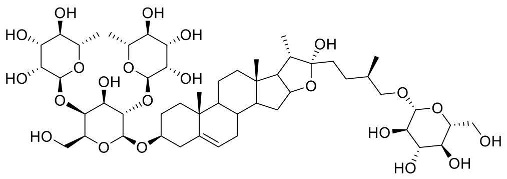

109 (30%)

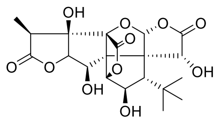

110 (14%)

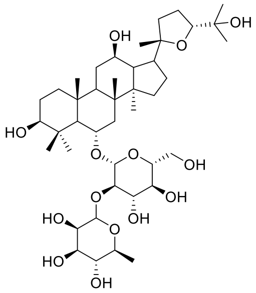

111 (32%)

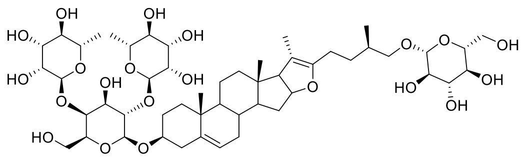

112 (25%)

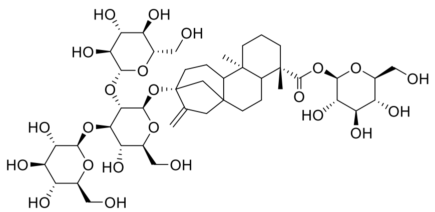

113 (17%)

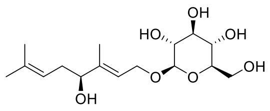

114 (32%)

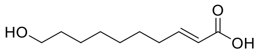

115 (21%)

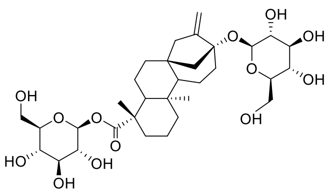

116 (13%)

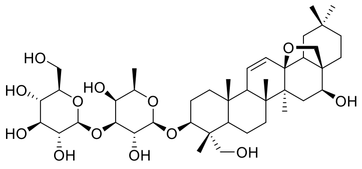

117 (16%)

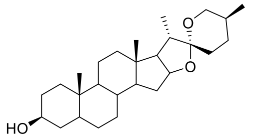

118 (14%)

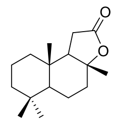

119 (10%)

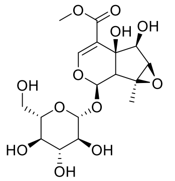

120 (32%)

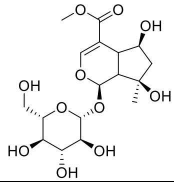

121 (16%)

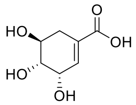

122 (27%)

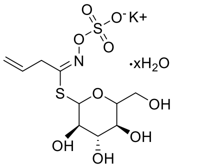

123 (26%)

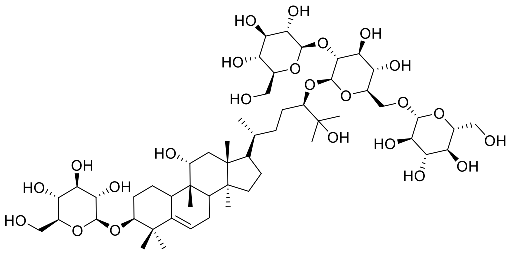

124 (37%)

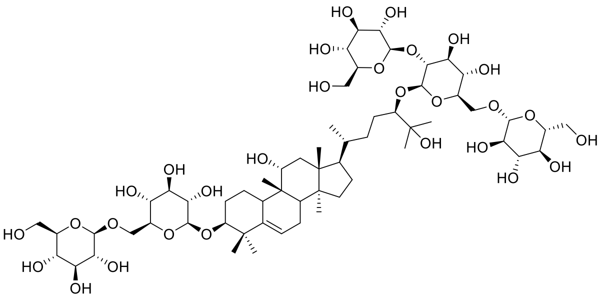

125 (12%)

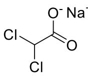

126 (18%)

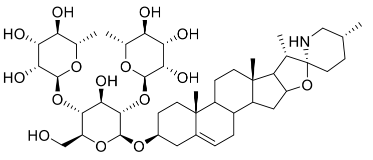

127 (-0.81%)

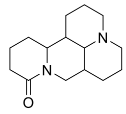

128 (41%)

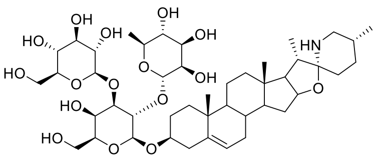

129 (24%)

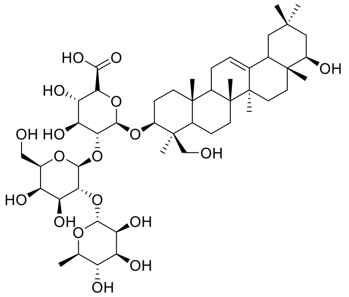

130 (-4.1%)

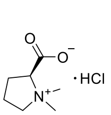

131 (33%)

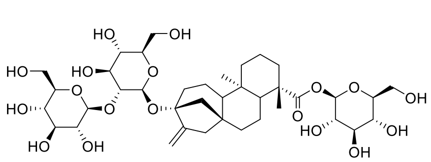

132 (33%)

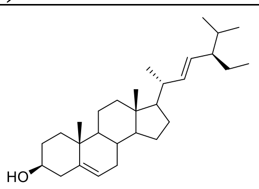

133 (56%)

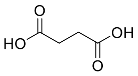	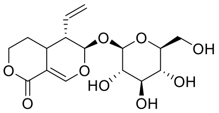	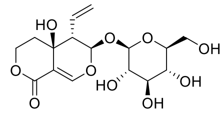	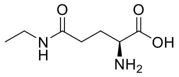
**134** (51%)	**135** (31%)	**136** (31%)	**137** (29%)
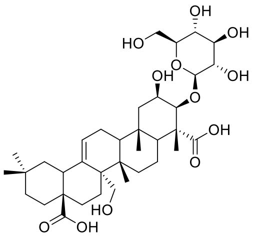	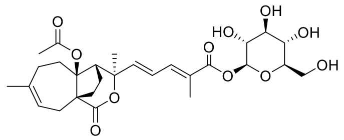	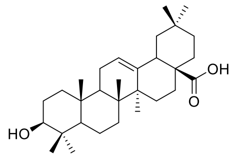	
**138** (19%)	**139** (104%)	**140** (9.9%)	
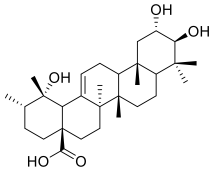	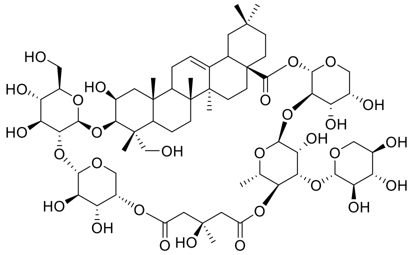	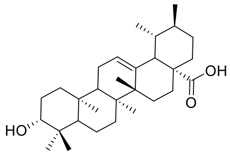	
**141** (23%)	**142** (33%)	**143** (3.6%)	
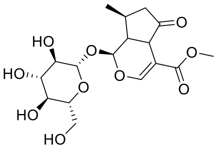	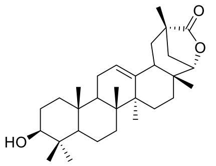		
**144** (38%)	**145** (20%)		

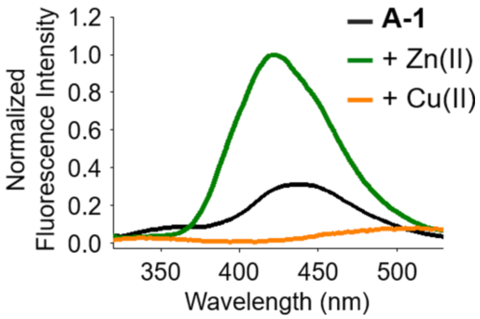

Fig. S1 Fluorescent response of **A-1** (black) upon treatment with Zn(II) (green) or Cu(II) (orange). Conditions: [**A-1**] = 500 nM; [$ZnCl_2$] = 100 μM; [$CuCl_2$] = 500 nM; 10% DPBS; λ_{ex} = 280 nm; λ_{em} = 420 nm; room temperature.

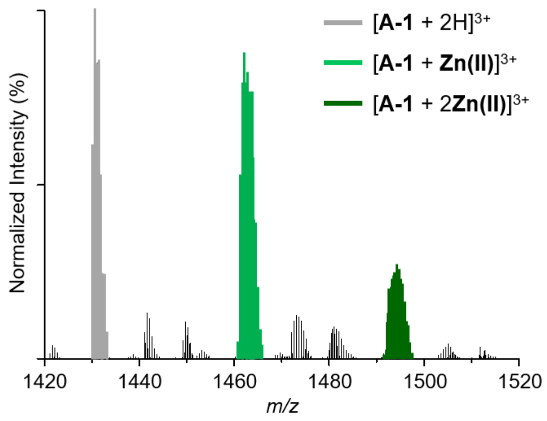

Fig. S2 Zn(II) binding of **A-1**, monitored by MS. Conditions: [**A-1**] = 20 μM; [$ZnCl_2$] = 2.0 mM; incubation for 1 h; 20 mM ammonium acetate, pH 7.4; room temperature; no agitation.

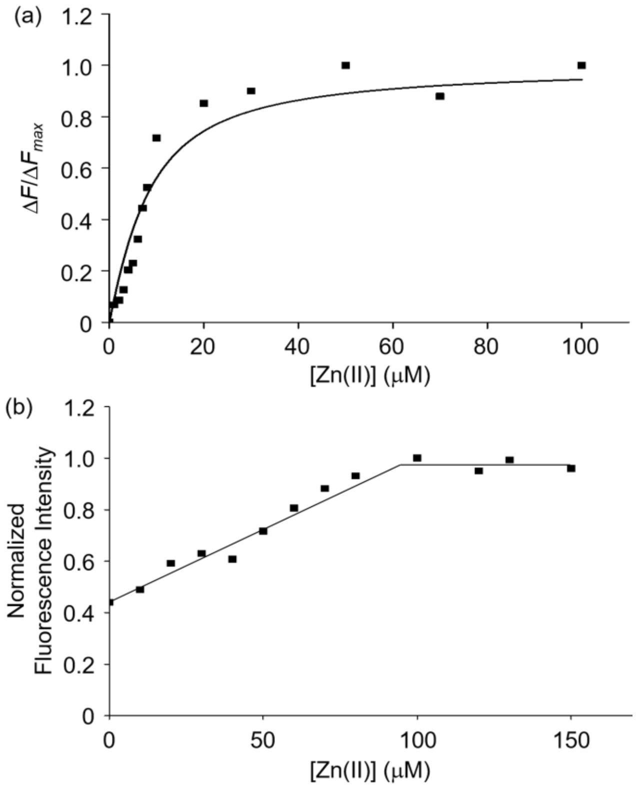

Fig. S3 Variation of the FRET intensity of **A-1** (at 420 nm) upon titration with Zn(II). (a) Plot of $\Delta F/\Delta F_{max}$ of Zn(II)-bound **A-1** (5 μM) as a function of Zn(II) concentration. (b) Change in the fluorescence intensity of **A-1** (500 nM) upon treatment with various concentrations of Zn(II) at 420 nm. Conditions: [**A-1**] = 5 μM or 500 nM; [$ZnCl_2$] = 0–100 or 0–150 μM; 10% DPBS; λ_{ex} = 280 nm; λ_{em} = 420 nm; room temperature.

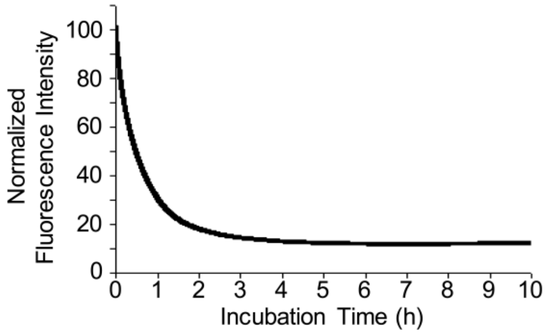

Fig. S4 Change in the FRET signal of **A-1** following the incubation time without Zn(II). Conditions: [**A-1**] = 300 nM; λ_{ex} = 280 nm; λ_{em} = 420 nm; incubation for 10 h; room temperature.

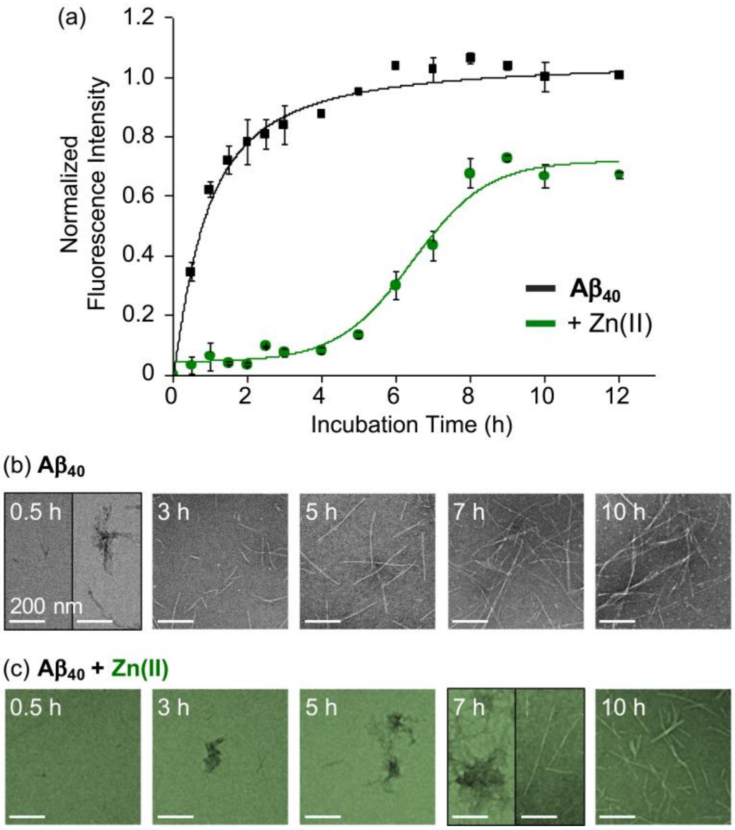

Fig. S5 Time-dependent aggregation progression of Aβ_{40} with and without Zn(II). (a) Aggregation kinetics of metal-free Aβ_{40} and Zn(II)–Aβ_{40}, observed by the ThT assay. Conditions: [Aβ_{40}] = 20 µM; [$ZnCl_2$] = 20 µM; [ThT] = 20 µM; pH 7.4; 37 °C; constant agitation. (b) Morphologies of Aβ_{40} aggregates generated at various incubation time points, visualized by TEM (scale bar = 200 nm). Conditions: [Aβ_{40}] = 20 µM; [$ZnCl_2$] = 20 µM; pH 7.4; 37 °C; constant agitation.

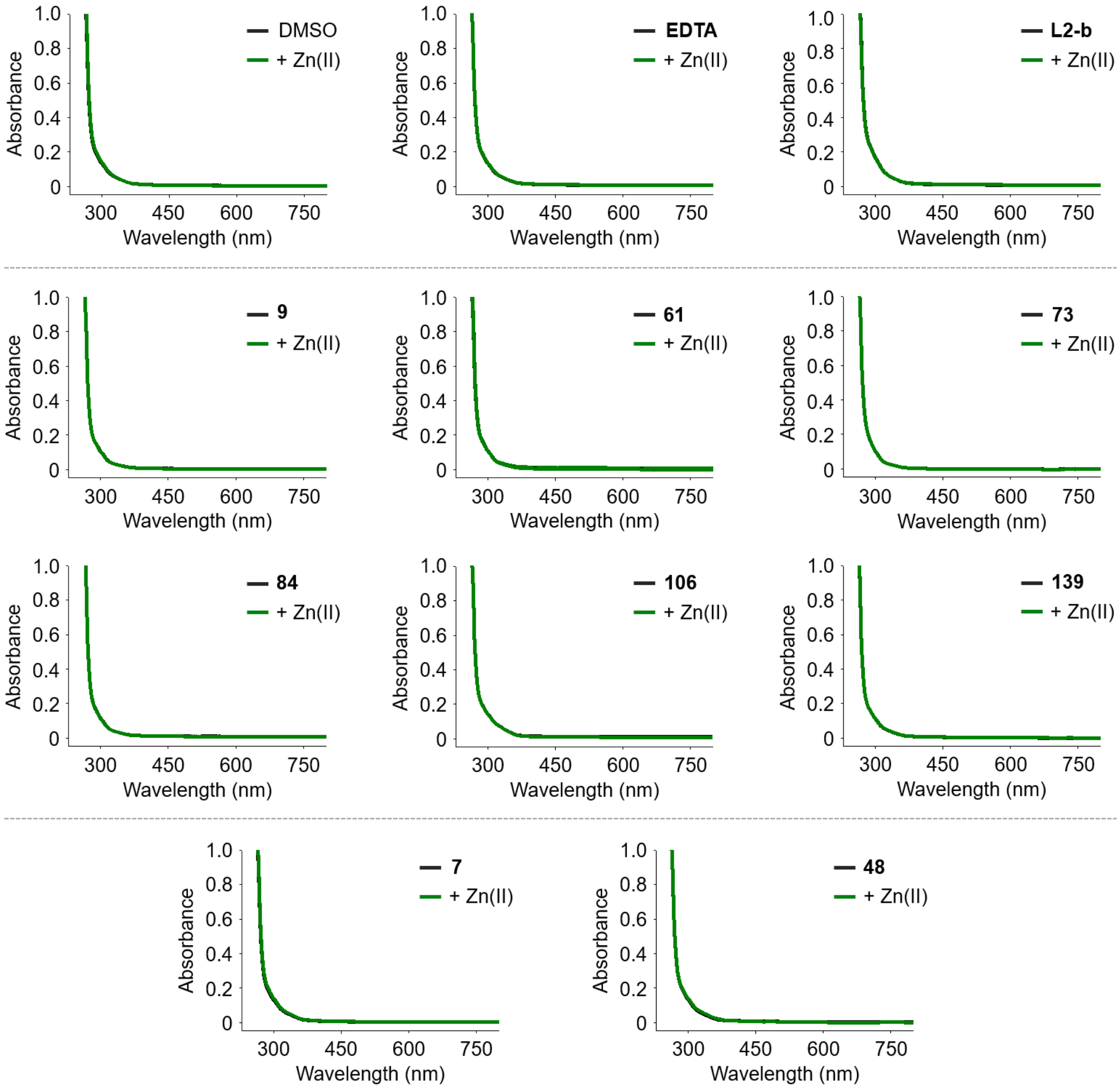

Fig. S6 Absorption spectra of **EDTA**, **L2-b**, and the selected natural products in the absence (black) and presence (green) of Zn(II). Conditions: [compound] = 100 μM; [$ZnCl_2$] = 100 μM. Note that the absorption spectra were obtained by a microplate reader.

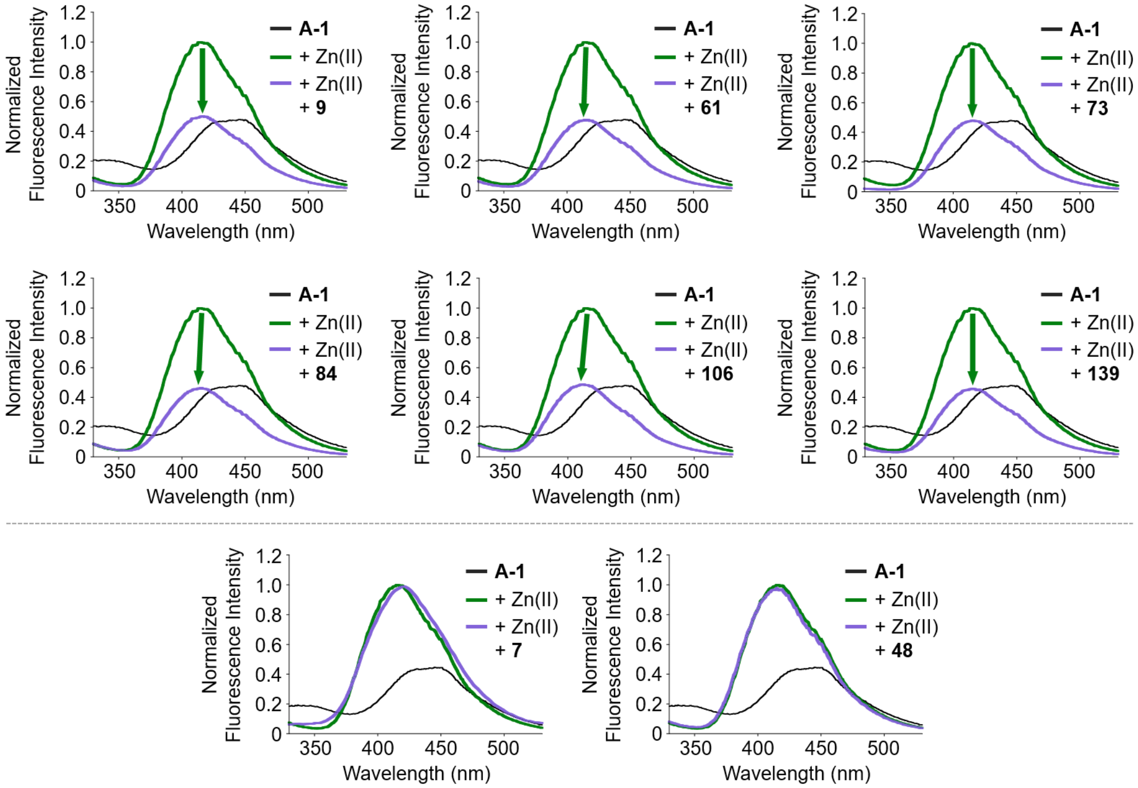

Fig. S7 FRET responses of **A-1** (black) to Zn(II) without (green) and with (purple) the selected natural products. The FRET intensities of the selected natural products showing noticeable inhibition (**9**, **61**, **73**, **84**, **106**, and **139**) or no significant inhibition (**7** and **48**) against Zn(II)–Aβ interaction were monitored. Conditions: [**A-1**] = 300 nM; [$ZnCl_2$] = 100 μM; [compound] = 100 μM; λ_{ex} = 280 nm.

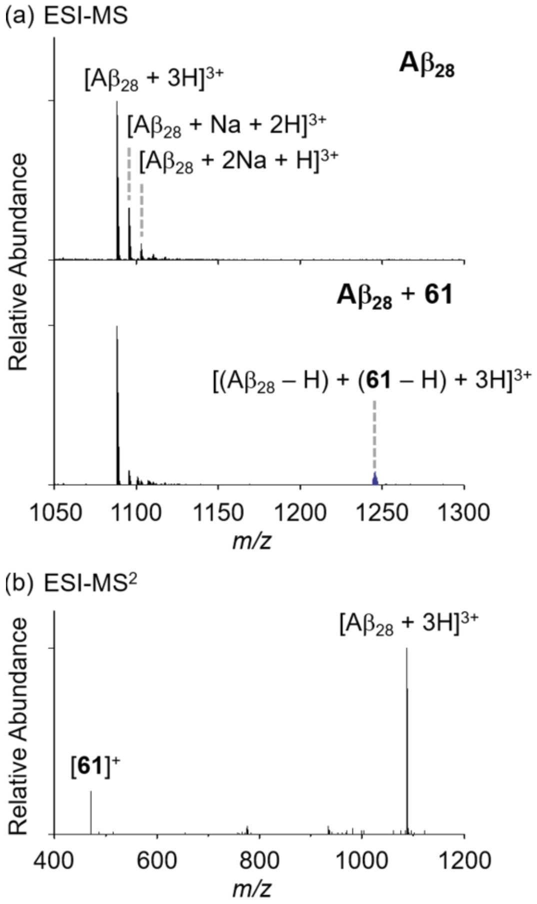

Fig. S8 Mass spectrometric analysis of the sample containing $A\beta_{28}$ with the natural product, **61**. (a) ESI-MS spectra of $A\beta_{28}$ incubated without (top) and with (bottom) **61**. (b) Tandem MS (ESI-MS^2) spectrum of the +3-charged $A\beta_{28}$–**61** complex (blue, $[(A\beta_{28} - H) + (\mathbf{61} - H) + 3H]^{3+}$, 1244 *m/z*). The ESI-MS^2 results support the formation of a covalent complex composed of $A\beta_{28}$ and **61**. Conditions: [Aβ] = 5 μM; [**61**] = 25 μM; 20 mM ammonium acetate, pH 7.4; incubation for 2 h; room temperature; no agitation.

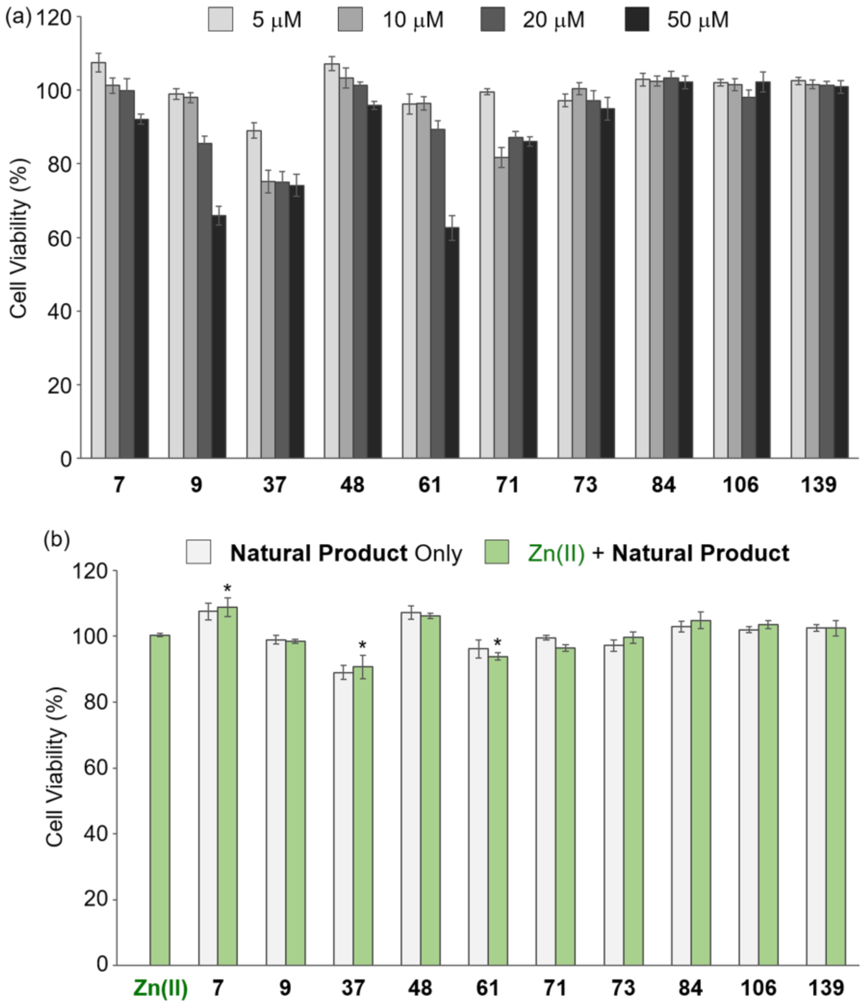

Fig. S9 Toxicity of the selected natural products with and without Zn(II) in 5Y cells. (a) Cells were treated with various concentrations (5, 10, 20, and 50 μM) of compounds for 24 h at 37 °C. (b) Compounds (5 μM) in the absence (gray) and presence (green) of Zn(II) (same equivalent to the compounds; 5 μM) were added to cells and incubated for 24 h at 37 °C. Cell viability (%) was determined by the MTT assay. The viability value was calculated compared to that of the cells added with DMSO only (1%, v/v). Error bars represent S.E.M. from three independent experiments. *$P < 0.05$ [*versus* Zn(II) or natural products].

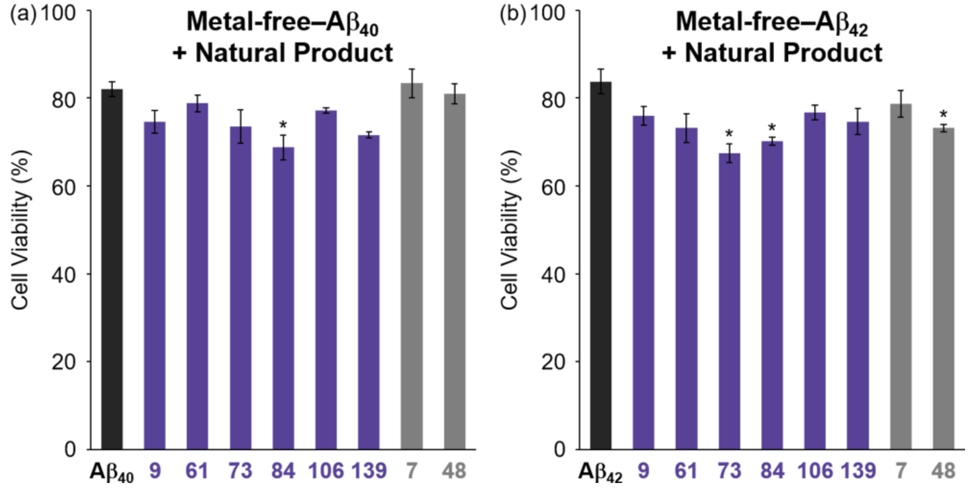

Fig. S10 Effect of the selected natural products on the toxicity triggered by metal-free Aβ in 5Y cells. (a) $A\beta_{40}$ or (b) $A\beta_{42}$ (10 μM) was pre-incubated at room temperature for 1 h and then treated to 5Y cells with compounds (10 μM) for 24 h. Cell viability (%) was determined by the MTT assay compared to that of the cells treated with a volume of H_2O (1% v/v DMSO) equal to the samples added. Error bars represent S.E.M. from three independent experiments. *$P < 0.05$.
